# GXYLT2 serves as a prognostic biomarker and is associated with β-catenin activation and gastric cancer aggressiveness

**DOI:** 10.1016/j.gendis.2025.101673

**Published:** 2025-05-05

**Authors:** Jiale Yang, Jiajun Wu, Ziqiang Chen, Xiangyun Hou, Xiaojing Li, Zhaorui Liu, Kai Yin, Tao Pang, Ruimin Huang, Jun Yan

**Affiliations:** a Center for Drug Safety Evaluation and Research, Shanghai Institute of Materia Medica, Chinese Academy of Sciences, Shanghai 201203, China; b University of Chinese Academy of Sciences, Beijing 100049, China; c State Key Laboratory of Genetic Engineering, School of Life Sciences, Fudan University, Shanghai 200438, China; d Department of Gastrointestinal Surgery, The First Affiliated Hospital of Naval Medical University, Shanghai 200433, China; e Laboratory Animal Center, Fudan University, Shanghai 200032, China

**Keywords:** Gastric cancer, GXYLT2, Prognostic biomarker, Tumor aggressiveness, Wnt/β-catenin signaling

## Abstract

Gastric cancer (GC) is a significant global health challenge due to its high incidence and mortality rate. However, the existing classification methods for GC still have limitations. Given the pivotal role of aberrant glycosylation in GC progression, there is a compelling need to develop a novel molecular classification for this disease. Using a comprehensive analysis of 186 glycogenes across seven public datasets encompassing 1547 GC patients, a 12-glycogene signature-based molecular classification was established, which was linked to tumor stage and prognosis. Among them, the overexpression of glucoside xylosyltransferase 2 (GXYLT2) was positively associated with tumor stage, diffuse subtype, and unfavorable survival outcomes in GC patients. Furthermore, GXYLT2 depletion significantly inhibited the proliferation, invasion, and sphere formation capacities in HGC-27, MKN1, and MKN45 GC cells with diffuse-subtype features, whereas its ectopic expression in AGS and MKN74 GC cells with intestinal subtype did not enhance their aggressive properties. Moreover, RNA sequencing analysis revealed that GXYLT2 knockdown resulted in the decrease of Wnt/β-catenin signaling, which was corroborated by TOPFlash reporter activity, β-catenin phosphorylation, immunofluorescence staining, and nuclear-cytoplasmic separation assays for its nuclear location, via the activation of PP2A complex dependent on GXYLT2-PP2A Aα interaction. Notably, GXYLT2 knockdown significantly suppressed tumorigenicity *in vivo*. Taken together, we identified GXYLT2 as a potential prognostic biomarker for GC patients, and targeting GXYLT2 suppressed the tumor aggressiveness and inhibited the Wnt/β-catenin pathway, which may provide a potential therapeutic target for GC patients.

## Introduction

Gastric cancer (GC) stands out as a prominent worldwide health challenge, marked by its high incidence and mortality rate.^1^^,^ ^2^ It manifests as a complex and heterogeneous disease, encompassing a variety of histological types, molecular profiles, and biological behaviors.^3^^,^ ^4^ Early-stage GC patients typically undergo endoscopic therapy, whereas those with advanced disease receive neoadjuvant therapy. Metastatic cases are generally treated with systemic anti-neoplastic agents, such as chemotherapy, targeted therapy, and immune checkpoint inhibitors.^5^ Given the conventional reliance on stage/grade for GC treatment, its efficacy is often constrained. Due to the high heterogeneity of GC, there is an urgent need for a more precise classification approach.

The existing pathological classifications for GC include several systems, such as the Nakamura classification, the World Health Organization (WHO) classification, and the Lauren classification.^6^, ^7^, ^8^ While the Nakamura classification distinguishes GC into differentiated and undifferentiated subtypes, the WHO classification further divides GC into tubular, papillary, mucinous, and poorly cohesive subtypes. The Lauren classification, on the other hand, categorizes GC based on histological features into intestinal, diffuse, and mixed subtypes.^9^ The intestinal and diffuse subtypes exhibit distinct clinical behaviors and molecular characteristics, resulting in different responses to chemotherapy.^10^ Specifically, the diffuse subtype is more aggressive and associated with a poorer prognosis compared with the intestinal subtype.^11^^,^ ^12^ This highlights the importance of accurate classification in guiding treatment strategies and predicting patient outcomes in GC.

Although these classification systems are instrumental in guiding treatment plans and assessing prognosis, they suffer from prognostic ambiguity, variable treatment responses, and limited predictive accuracy.^3^ For instance, the Lauren classification requires invasive sampling and has been noted for its limited accuracy,^13^ with a reported consistency between biopsy and surgical specimens of only 64.7%.^14^ The WHO classification is also criticized for being overly complex. Given these limitations, there is a pressing need for more refined classification approaches that leverage molecular markers to improve clinical practice.

Current molecular classification mainly encompasses The Cancer Genome Atlas (TCGA) classification, “Singapore-Duke” classification, “Mesenchymal” classification, Asian Cancer Research Group (ACRG) classification, and “Intrinsic” classification.^15^, ^16^, ^17^, ^18^, ^19^ i) The TCGA classification identifies four subtypes: Epstein–Barr virus (∼15%), microsatellite instability (∼26%), chromosomal instability (∼23%), and genetically stable (∼36%), with the latter being linked to the most adverse prognosis. ii) The “Singapore-Duke” system categorizes GC into proliferative, metabolic, and mesenchymal subtypes, with the mesenchymal subtype being associated with the poorest prognosis. iii) The “Mesenchymal” classification divides GC into epithelial and mesenchymal subtypes, identifying the mesenchymal subtype as having a more unfavorable prognosis. iv) The ACRG classification segments GC into microsatellite instability, microsatellite stable (MSS) with epithelial–mesenchymal transition (EMT), MSS with TP53 mutation (MSS/TP53^+^), and MSS without TP53 mutation (MSS/TP53^−^) subtypes, with the EMT subtype being associated with the worst prognosis. v) The “Intrinsic” classification categorizes GC into genomic intestinal (G-INT) and diffuse (G-DIF) subtypes, which correspond to the intestinal and diffuse subtypes as defined by the Lauren classification, respectively. However, the high cost of omics-based assays, the complexity of bioinformatic analyses, along with the highly heterogeneous nature of GC, have hampered the clinical applications of the above molecular classification.^20^, ^21^, ^22^ As a result, the novel biomarkers and classification approaches are thus urgently needed for GC therapy.

Glycosylation, a vital post-translational modification of proteins, is essential to numerous cellular processes, such as protein folding, transport, and function.^23^ It is particularly critical in GC pathogenesis. For example, abnormal O-linked and N-linked glycosylation modifications, mediated by enzymes such as N-acetylgalactosaminyl-transferase 1 (GALNT1) and ST6 beta-galactoside alpha-2,6-sialyltransferase 1 (ST6GAL1), have been linked to the activation of the Wnt/β-catenin pathway and the development of trastuzumab-resistance in GC cells, respectively.^24^^,^^25^ These findings underscore the potential of glycosylation in modulating cancer progression and therapeutic responses.

Since we and others have successfully exploited glycogene-based classification to molecularly stratify bladder cancer and colon cancer,^26^^,^^27^ we in this study analyzed the mRNA expression profiling and clinical relevance of glycogenes across multiple public GC datasets, to explore the advantage of glycogene-based molecular classification and to pinpoint a glycogene-derived prognostic biomarker for GC. In addition, we explored the potential mechanism by which this prognostic biomarker might contribute to GC aggressiveness.

## Materials and methods

Detailed information can be found in Supplementary data. It includes experimental methods, such as western blotting assay, immunohistochemistry staining, RNA sequencing analysis, and tumorigenicity study. Antibody information (Table S1), the sequences of reverse transcription PCR and shGXYLT2 (Table S2), and information of kits and reagents (Table S3) are included in Doc S1.

### Data collection

Gene expression data and clinical information of TCGA-STAD (TCGA stomach cancer) and TCGA (Nature 2014) were obtained from the Genomic Data Commons Data Portal (https://portal.gdc.cancer.gov) and Cbioportal (https://www.cbioportal.org), respectively. Other gene expression data and clinical information of GC patients were obtained from the Gene Expression Omnibus (GEO) database (http://www.ncbi.nlm.nih.gov/geo). Gene expression data of GC cell lines were obtained from the Cancer Cell Line Encyclopedia (CCLE) database (https://sites.broadinstitute.org/ccle). For unsupervised hierarchical clustering, FPKM (fragments per kilobase of transcript per million fragments mapped) was log-transformed (Log_2_(1+FPKM)) and normalized using Z-score normalization (mean-centered). In cases where a gene had multiple probes, the averaged expression values were used. A total of 186 glycogenes were obtained from the glycogene database (GGDB, https://acgg.asia/ggdb2).**^26^**

### Bioinformatics analyses for public data from GC patients

Unsupervised hierarchical clustering of TCGA-STAD and GEO datasets was indicated in Morpheus (https://software.broadinstitute.org/morpheus) by the average linkage method with one minus Pearson correlation. Differentially expressed glycogenes in the TCGA-STAD dataset were defined as |fold change| ≥ 2, *p* < 0.05, and false discovery rate < 0.05, and further analyzed using Limma, an R package, based on transcripts per million.

To explore the relationship between glycogenes and molecular features, data from seven independent datasets (TCGA-STAD, GSE15459, GSE62254, GSE26901, GSE84426, GSE84433, and GSE26899; *n* = 1547 in total) were analyzed using Gene Set Variation Analysis (GSVA).**^28^** GSVA scores were calculated using the GSVA package of R software (version 4.0.2). Pearson correlation coefficients were used to determine the correlations between gene expression levels and molecular features, and *p*-values were assessed using the *t*-test. The gene sets, including G-DIF markers, G-INT markers, and up-regulated and down-regulated EMT (EMT-up and EMT-down) genes, were referred to the previous literatures.^16^^,^^19^

### Human GC specimens

The study was approved by the Ethics Committee of the First Affiliated Hospital of Naval Medical University and conducted in accordance with the Declaration of Helsinki. The written informed consents had been obtained from all corresponding patients. Tissue paraffin sections from 42 GC patients, including 22 intestinal-type and 20 diffuse-type cases, were obtained for immunohistochemistry staining.

### Luciferase activity assay

To validate the activation of Wnt/β-catenin signaling, activity of TOPFlash (TCF reporter plasmid) or FOPFlash (negative control) was examined by a dual luciferase reporter assay kit (RG027; Beyotime), according to the manufacturer's instructions. In brief, 293T cells (3 × 10^5^ cells per well) were seeded into a 6-well plate and transfected with a mixture of TOPFlash or FOPFlash and pRL-TK plasmid (1 μg: 0.1 μg). 48 h post-transfection, all groups were incubated in serum-free medium for 24 h. Samples were then collected and analyzed following the kit instructions. The firefly luciferase activity was normalized to Renilla luciferase activity, and the TOP/FOP ratio was used to measure the β-catenin-driven transcription.

### Nuclear and cytoplasmic protein extraction

1–1.5 × 10^6^ cells/well were plated in a 10 cm dish. After HGC-27 cells were serum-starved for 48 h, nuclear and cytoplasmic proteins were isolated using a nuclear and cytoplasmic protein extraction kit (P0028; Beyotime Biotechnology), according to the manufacturer's instructions.

### Statistical analyses

Data were presented as mean ± standard deviation. The *χ*^2^ test was used for the correlation between glycogene-based classification and other classifications. The two-tailed unpaired student's *t*-test was used for the correlation between the 12-glycogene signature and GSVA score of EMT-up/EMT-down or G-DIF/G-INT feature. Univariate and multivariate models were calculated using Cox proportional hazards regression in the R packages “survival” and “survminer”. Survival probabilities were assessed by Kaplan–Meier analysis with the log-rank test. Two-tailed unpaired student's *t*-test was used to analyze differences between variables. ANOVA was used to analyze the difference between the means of more than two groups; one-way ANOVA involves one independent variable, while two-way ANOVA involves two independent variables. Statistical significance was set as *p* < 0.05. GraphPad Prism 8.0 or R 4.0.2 was utilized for statistical analyses.

## Results

### Molecular classification of GC based on mRNA expression levels of glycogenes

**Figure 1:**
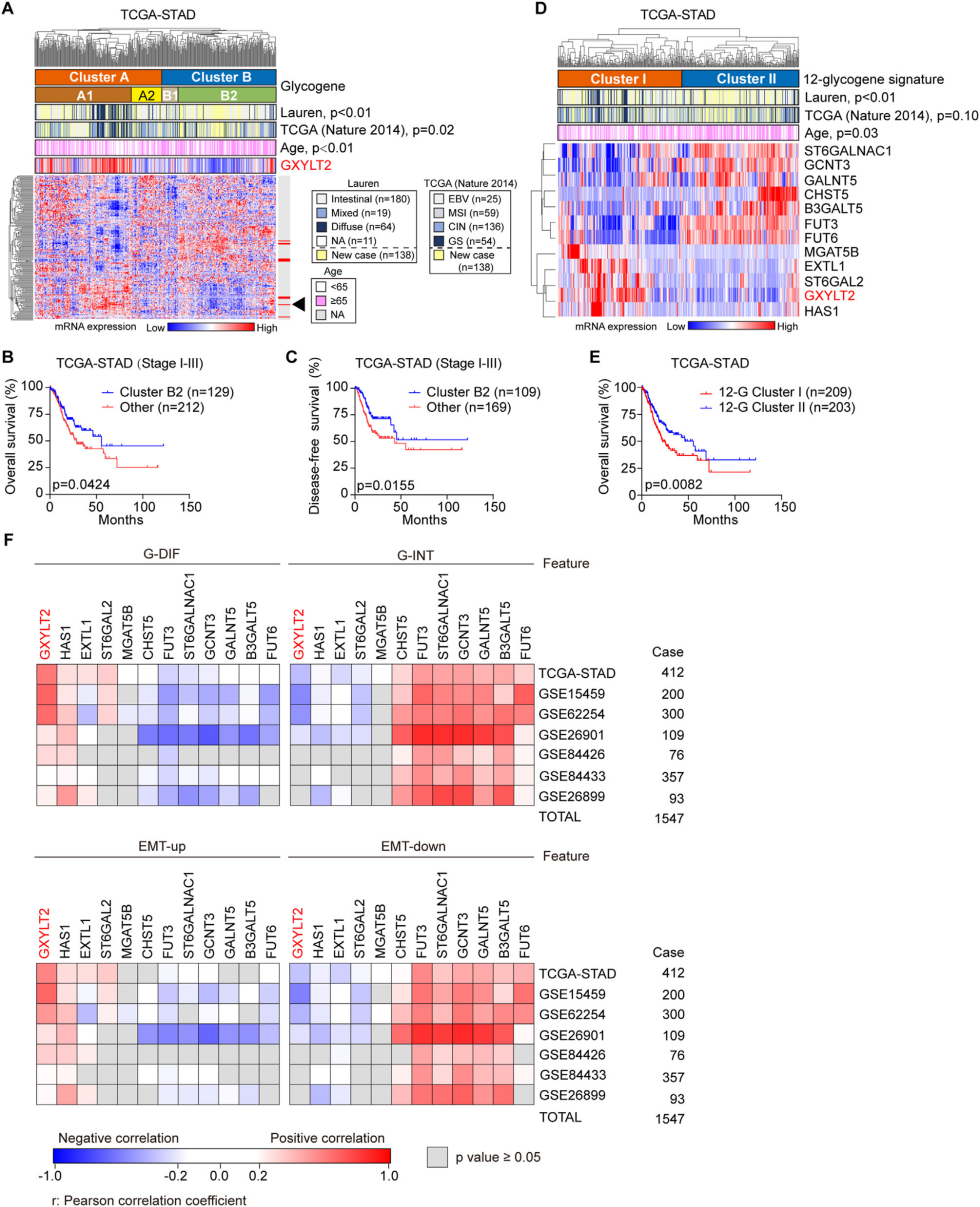
Identification of glycogene-based molecular classification and correlations between the expression levels of 12-glycogene signature and molecular features in GC patients. **(A)** Unsupervised clustering of 186 glycogenes in the TCGA-STAD cohort (*n* = 412) separated GC patients into two clusters (A and B) and four subclusters (A1, A2, B1, and B2). The annotations by the Lauren classification (Intestinal, Diffuse, Mixed, and NA; *n* = 274), the TCGA (Nature 2014) classification (EBV, CIN, MSI, and GS; *n* = 274), and patients' age were respectively indicated for the same GC patient. The 138 new GC cases without Lauren or TCGA classification information were defined as “new case” (in yellow). The black arrowhead indicates GXYLT2. **(B, C)** Kaplan–Meier plots for overall survival (B) and disease-free survival (C) in GC patients at stages I-III from the TCGA-STAD dataset, comparing that in subcluster B2 with the other three subclusters (A1, A2, and B1). **(D)** Clustering based on 12-glycogene signature in GC patients from the TCGA-STAD cohort (*n* = 412). Two clusters (I and II) were compared with the Lauren classification, the TCGA (Nature 2014) classification, and patients' age. **(E)** Kaplan–Meier plot for overall survival in GC patients from the TCGA-STAD dataset, stratified by 12-glycogene signature classification as cluster I and cluster II. **(F)** Pearson correlation coefficients (r) for the correlations between the expression levels of the 12 glycogenes and molecular features, including G-DIF, G-INT, EMT-up, and EMT-down, across seven independent cohorts (*n* = 1547 total). The heatmap was composed of color-coded blocks: red, *r* > 0.25, *p* < 0.05; blue, *r* < −0.25, *p* < 0.05; white, |r| ≤ 0.25, *p* < 0.05; gray, *p* ≥ 0.05. *p* values were calculated with the *χ*^2^ test (A and D). GC, gastric cancer; TCGA-STAD, TCGA (stomach cancer); G-INT, genomic intestinal; G-DIF, genomic diffuse; EMT, epithelial–mesenchymal transition. EBV, Epstein–Barr virus; MSI, microsatellite instability; CIN, chromosomal instability; GS, genetically stable; EMT-up, up**-**regulated EMT; EMT-down, down**-**regulated EMT; GXYLT2, glucoside xylosyltransferase 2.

To investigate whether aberrant glycogene expression could define molecular subtypes of GC, we analyzed the transcriptomic data from public GC databases. Unsupervised hierarchical clustering of 186 unique glycogenes in GC patients from TCGA-STAD dataset (*n* = 412) unveiled two principal clusters (labeled as A and B) and further delineated four subclusters (A1, A2, B1, and B2; Fig. 1A). This glycogene-based molecular classification was juxtaposed with the Lauren classification and the TCGA classification, respectively. Because the Lauren classification and the TCGA classification information were obtained from a paper published in 2014,^15^ only 274 out of 412 GC patients with the classification definition, the new 138 GC patients in the TCGA-STAD dataset were defined as “new case”. Interestingly, diffuse and intestinal subtypes in the Lauren classification were respectively enriched in subcluster A1 and subcluster B2 of our classification (*p* < 0.01; Fig. 1A); the most aggressive genetically stable subtype in the TCGA classification was enriched in subcluster A1, as well as the more favorable cases were linked to the B2 subcluster (*p* = 0.02; Fig. 1A). We further investigated the relationship between this glycogene-based molecular classification and patients' prognosis. In GC patients at stage I-III, both overall survival (*p* = 0.0424; Fig. 1B) and disease-free survival (*p* = 0.0155; Fig. 1C) in subcluster B2 were significantly better than those in other subclusters (A1, A2, and B1 in combination); when we compared the outcomes in the four subclusters individually, subcluster B2 was only linked to superior disease-free survival outcome over subcluster B1 (*p* < 0.001; Fig. S1). In summary, this glycogene-based molecular classification not only demonstrated its relevance with current pathological and molecular GC classifications but also underscored its prognostic significance in GC patients, offering a promising avenue for more personalized treatment strategies.

The most representative differentially expressed glycogenes between B2 and other subclusters were identified, including GXYLT2, HAS1, EXTL1, ST6GAL2, MGAT5B, CHST5, FUT3, ST6GALNAC1, GCNT3, GALNT5, B3GALT5, and FUT6 in TCGA-STAD dataset (*p* < 0.01; Table S4). Interestingly, this 12-glycogene signature could successfully divide the GC patients in TCGA-STAD dataset into two clusters (cluster I and II; Fig. 1D). Similarly, diffuse subtype and intestinal subtype in the Lauren classification were enriched in cluster I and II, respectively (*p* < 0.01; Fig. 1D). The overall survival of cluster I (*n* = 209) was significantly shorter than that of cluster II (*n* = 203; *p* = 0.0082; Fig. 1E; with hazard ratio (HR) (95% confidence interval (CI)) = 0.645 (0.469–0.888), *p* = 0.0071, by univariate analysis; Table S5). The disease-free survival of cluster I (*n* = 156) was significantly shorter than that of cluster II (*n* = 164; *p* = 0.0468; Fig. S2A; with HR (95% CI) = 0.680 (0.464–0.997), *p* = 0.0481, by univariate analysis; Table S5). The 12-glycogene signature for GC classification was validated using three additional GC datasets: GSE15459 (*n* = 200), GSE26901 (*n* = 109), and GSE62254 (*n* = 300). In GSE15459, the significant correlations were observed between 12-glycogene signature classification and the Lauren classification (*p* = 0.02) or the “Singapore-Duke” classification (*p* < 0.01). The invasive subtype with poor prognosis by the “Singapore-Duke” classification was highly enriched in cluster I (Fig. S3A). In GSE26901, 12-glycogene signature classification was only significantly associated with the “Mesenchymal” classification (*p* < 0.01), and the mesenchymal subtype therein with poor prognosis^18^ was also enriched in cluster I (Fig. S3C). In GSE62254, 12-glycogene signature classification was only significantly correlated to the ACRG classification (*p* < 0.01), with the enrichment of EMT subtype (with poor prognosis)^17^^,^^29^ in cluster I (Fig. S3E). Furthermore, GC patients classified into cluster I and II by the 12-glycogene signature respectively exhibited poor and favorable overall survival across three datasets (*p* < 0.01; Figs. S3B, D, F). The univariate analysis for overall survival showed HR (95% CI) = 0.576 (0.381–0.871), *p* = 0.0088 in GSE15459 (Table S6); HR (95% CI) = 0.490 (0.285–0.842), *p* = 0.0098 in GSE26901 (Table S7); and HR (95% CI) = 0.461 (0.333–0.637), *p* < 0.0001 in GSE62254 (Table S8). Consistently, in the GSE62254 dataset, the disease-free survival of cluster I was significantly shorter than that of cluster II (*p* < 0.0001; Fig. S2B; with HR (95% CI) = 0.420 (0.294–0.601), *p* < 0.0001, by univariate analysis; Table S8).

The associations between the 12-glycogene signature and molecular features, including G-DIF/G-INT by the “Intrinsic” classification^19^ and EMT-up/EMT-down markers,^16^ were analyzed using mRNA levels of these 12 glycogenes and the GSVA scores of these four features in seven independent GC datasets (*n* = 1547 in total). Notably, among 12 glycogenes, glucoside xylosyltransferase 2 (GXYLT2) was the most prominent gene with strong positive correlations with G-DIF and EMT-up features, and negative correlations with G-INT and EMT-down features in most datasets, using Pearson correlation analysis (Fig. 1F). Thus, GXYLT2, as a potential prognostic biomarker, was suggested for GC patients.

### GXYLT2 as a potential prognostic biomarker for GC patients

**Figure 2:**
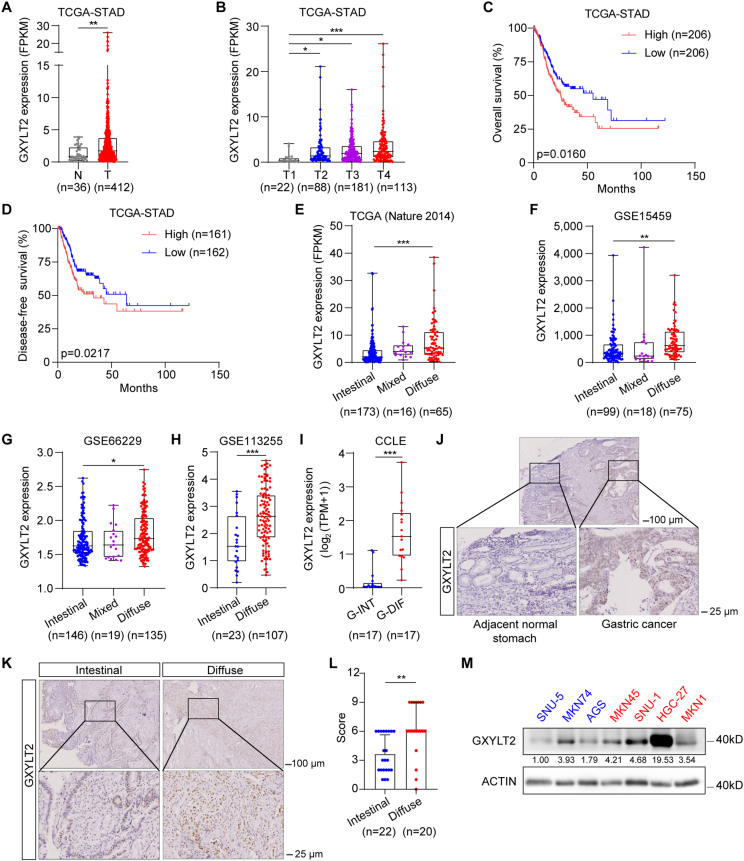
The correlation between GXYLT2 expression and clinical characteristics in GC patients. **(A, B)** GXYLT2 mRNA levels in GC and normal gastric tissues (A) and the tumor stage of GC patients (B) from the TCGA-STAD dataset. **(C, D)** Kaplan–Meier analysis of overall survival (C) and disease-free survival (D) of GC patients from the TCGA-STAD dataset. **(E**–**H)** GXYLT2 mRNA levels in three subtypes (Intestinal, Diffuse, and Mixed) by Lauren classification in GC patients from TCGA (Nature 2014) (E), GSE15459 (F), GSE66229 (G), and GSE113255 (H) datasets. **(I)** GXYLT2 mRNA levels in G-INT and G-DIF subtypes by “Intrinsic” classification in GC samples from the CCLE dataset. **(J, K)** Representative immunohistochemistry images for GXYLT2 protein expression in GC samples from our own cohort containing non-cancerous tissue (J) and the intestinal and diffuse subtypes (K). **(L)** Immunohistochemistry scores of GXYLT2 protein in the intestinal and diffuse subtypes from GC samples from our own cohort. **(M)** GXYLT2 protein expression in GC cell lines by western blotting assay. β-actin was used as the loading control. Blue, intestinal or G-INT subtype; red, diffuse or G-DIF subtype. Data were presented as mean ± standard deviation of three independent experiments. *p*-values were calculated with two-tailed unpaired student's *t*-test (A, H, I, and L) and one-way ANOVA (B, E, F, and G). ∗*p* < 0.05, ∗∗*p* < 0.01, and ∗∗∗*p* < 0.001. GXYLT2, glucoside xylosyltransferase 2; GC, gastric cancer; G-INT, genomic intestinal; G-DIF, genomic diffuse.

We analyzed GXYLT2 mRNA levels in GC samples with different clinical characteristics from five GC public datasets. GXYLT2 was overexpressed in GC tissues compared with the normal gastric tissues in TCGA-STAD and GSE113255 datasets (*p* < 0.01; Fig. 2A; Fig. S4A). GXYLT2 was significantly higher in GC tissues with high stages than those with low stages (*p* < 0.05, T2, T3, T4 *vs.* T1, in TCGA-STAD; *p* < 0.05, III-IV *vs.* I-II, in GSE66229) (Fig. 2B; Fig. S4B). It was also markedly increased in GC patients with recurrence in the GSE66229 dataset (*p* < 0.001; Fig. S4C). Kaplan–Meier analysis showed that higher GXYLT2 expression was significantly associated with lower overall survival in TCGA-STAD (*p* < 0.05; Fig. 2C), GSE15459 (*p* < 0.001; Fig. S4D), and GSE62254 datasets (*p* < 0.001; Fig. S4E), along with lower disease-free survival in TCGA-STAD (*p* < 0.05; Fig. 2D) and GSE62254 datasets (*p* < 0.001; Fig. S4F), respectively. These findings from public datasets indicated that GXYLT2 mRNA expression was positively correlated with tumor stage, tumor recurrence, and poor prognosis in GC patients.

Since clinicopathological correlation analyses showed that the patients' age (65-year-old as the cutoff value) was significantly associated with B2 subcluster (*p* = 0.0039; Table 1), as well as with our glycogene-based molecular classification (*p* < 0.01; Fig. 1A) in TCGA-STAD dataset, whether the alterations in glycosylation profiles and GXYLT expression were related to age was further investigated. The correlation analyses between age and the molecular classification based on 12-glycogene signature were conducted in TCGA-STAD, GSE15459, GSE26901, and GSE62254 datasets (Fig. 1D; Fig. S3A, C, E). Notably, the significant correlation was only observed in the TCGA-STAD dataset (*p* = 0.03). The changes in GXYLT2 mRNA levels across different tumor stages, tumor grades, family history of GC, and *H. pylori* infection status were analyzed based on patients' age in the TCGA-STAD dataset (Fig. S4G–J). Comparing GC patients older than 65 years old with the ones less than 65 years old, GXYLT2 was only up-regulated in the grade 3 population (*p* < 0.05) and only down-regulated in the population without a family history of GC (*p* < 0.05).

**Table 1:** The clinicopathological associations among 4 subclusters by glycogene-based molecular classification in TCGA-STAD dataset.

	Total (*n* = 412)	A1 (*n* = 166)	A2 (*n* = 54)	B1 (*n* = 23)	B2 (*n* = 169)	B2 *vs* A1 *p value*	B2 *vs* A2 *p value*	B2 *vs* B1 *p value*	B2 *vs* Others *p value*
Age (years)						0.0026	0.0611	0.9363	0.0039
< 65	173	83	26	8	56				
≥ 65	234	82	28	15	109				
NA	5	1	0	0	4				
Gender						0.2179	0.0992	0.1908	0.0739
Male	145	56	15	6	68				
Female	267	110	39	17	101				
Grade						0.0017	0.6437	0.0934	0.0034
G1	12	4	1	0	7				
G2	148	44	23	6	75				
G3	243	112	30	17	84				
NA	9	6	0	0	3				
Stage						0.0698	0.4580	0.1398	0.1261
I	58	19	8	2	29				
II	122	46	21	9	46				
III	169	83	15	12	59				
IV	39	13	7	0	19				
NA	24	5	3	0	16				

Notes: “Others” included A1, A2, and B1 subclusters.

In addition, GXYLT2 was up-regulated in the diffuse subtype compared with the intestinal subtype (*p* < 0.05) in TCGA-STAD, GSE15459, GSE66229, and GSE113255 datasets (Fig. 2E–H); and it was also higher in the G-DIF subtype than the G-INT subtype cell lines from CCLE^30^ (*p* < 0.001; Fig. 2I). Immunohistochemistry staining for GXYLT2 protein was performed in 42 GC samples from our own cohort (Table S9). On a representative GC tissue section containing both cancerous and normal gastric components, GXYLT2 exhibited strong signal intensities in the tumor lesion, but very weak signal in normal gastric tissue (Fig. 2J). We also observed that GXYLT2 protein levels were relatively low in the intestinal subtype (*n* = 22) and high in the diffuse subtype (*n* = 20; Fig. 2K), and these differences were quantified by immunohistochemistry score (*p* < 0.01; Fig. 2L). GXYLT2 protein levels were then examined by western blotting assay in seven GC cell lines (Fig. 2M). The diffuse subtype GC cell lines, especially HGC-27 cells, expressed a relatively higher level of GXYLT2, compared with the intestinal subtype, especially AGS and SNU5 cells.^19^^,^ ^31^^,^ ^32^ In summary, GXYLT2 overexpression was associated with the diffuse subtype of GC and served as a prognostic biomarker of GC.

### GXYLT2 knockdown suppressed GC cell proliferation, invasion, and sphere formation

**Figure 3:**
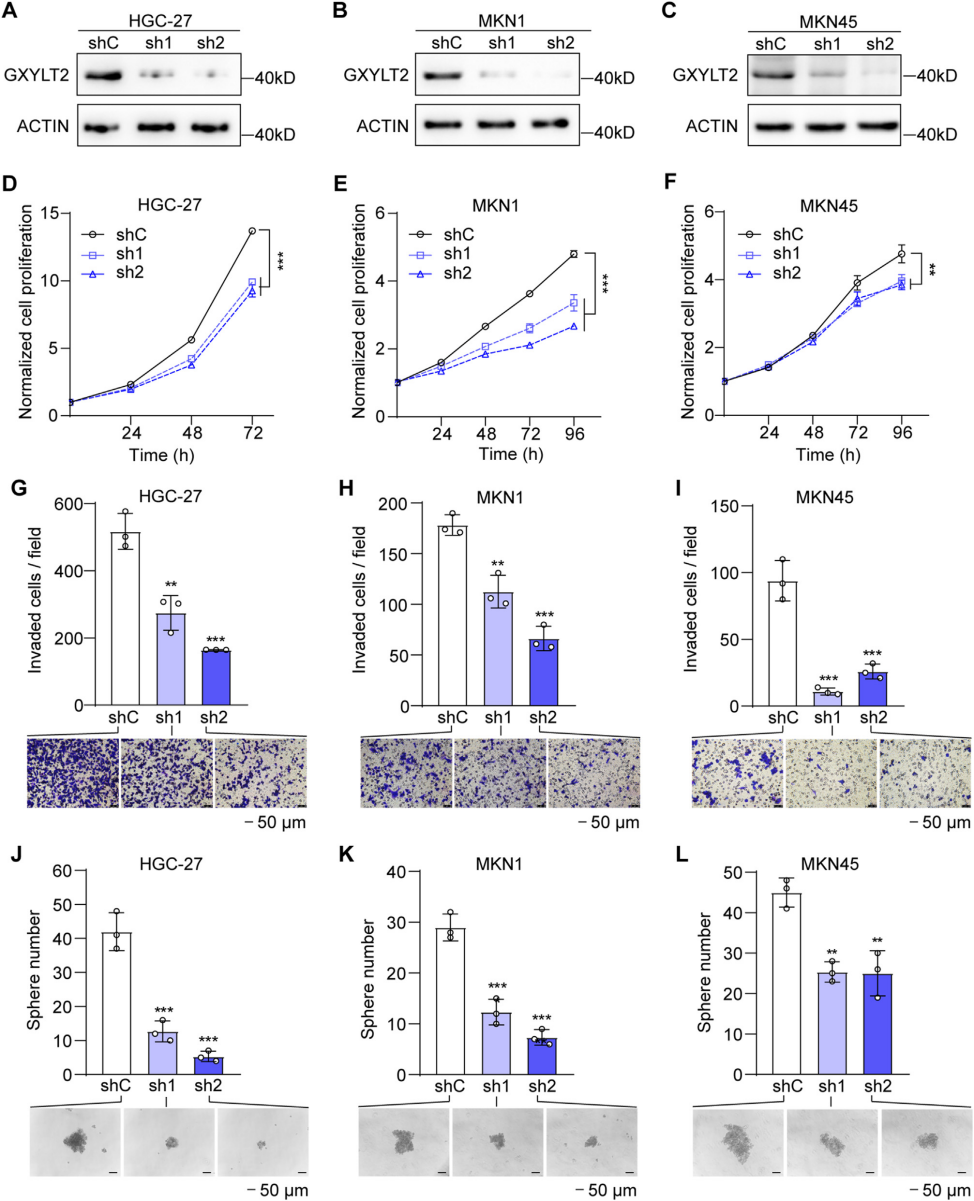
GXYLT2 knockdown suppressed the proliferation, invasion, and sphere formation of diffuse-subtype GC cells. **(A**–**C)** GXYLT2 knockdown by shRNAs (sh1 or sh2) in HGC-27 (A), MKN1 (B), and MKN45 (C) cells, detected by western blotting analysis. shC, control cells. **(D**–**F)** The proliferation of GXYLT2-knockdown HGC-27 (D), MKN1 (E), and MKN45 (F) cells by SRB assay. **(G**–**I)** The invasion of GXYLT2-knockdown HGC-27 (G), MKN1 (H), and MKN45 (I) cells by transwell assay. **(J**–**L)** Sphere formation ability of GXYLT2-knockdown HGC-27 (J), MKN1 (K), and MKN45 (L) cells. Scale bar, 50 μm. Data were presented as mean ± standard deviation of three independent experiments. *p*-values were calculated with two-way ANOVA (D–F) and one-way ANOVA (G–L). ∗∗*p* < 0.01 and ∗∗∗*p* < 0.001. GXYLT2, glucoside xylosyltransferase 2; GC, gastric cancer.

To dissect the role of GXYLT2 in GC cells, we stably knocked down its expression in the diffuse-subtype GC cell lines, including HGC-27, MKN1, and MKN45 cells, by two shRNAs targeting different regions of GXYLT2 mRNA (sh1 and sh2; Fig. 3A–C). GXYLT2 depletion not only significantly reduced cancer cell proliferation (Fig. 3D–F) but also suppressed the cell invasiveness of all three GC cell lines (Fig. 3G–I). Additionally, sphere formation assay revealed that GXYLT2 knockdown decreased sphere-formation capability of all three GC cell lines (*p* < 0.01; Fig. 3J–L). Interestingly, the depletion of GXYLT2 in the intestinal-subtype MKN74 cells had no significant effects on the proliferation, invasion, and sphere formation of cancer cells (Fig. S5A–D). Altogether, these results indicated that GXYLT2 was required for the proliferation, invasion, and sphere-forming capability in diffuse-subtype GC cells, but not in intestinal-subtype cancer cells.

On the other hand, FLAG-tagged GXYLT2 was re-introduced into AGS and MKN74 GC cells with intestinal-subtype traits (Fig. S6A). However, neither cell proliferation nor cell invasiveness exhibited significant changes upon GXYLT2 overexpression in both cell lines (Fig. S6B–E). Similarly, sphere formation assay showed that GXYLT2 overexpression did not significantly affect the sphere formation of AGS and MKN74 cells (Fig. S6F, G). Overall, these findings indicated that GXYLT2 overexpression was a necessary but not sufficient condition for GC aggressiveness.

### GXYLT2 knockdown repressed the expression of Wnt signaling-related genes in GC cells

**Figure 4:**
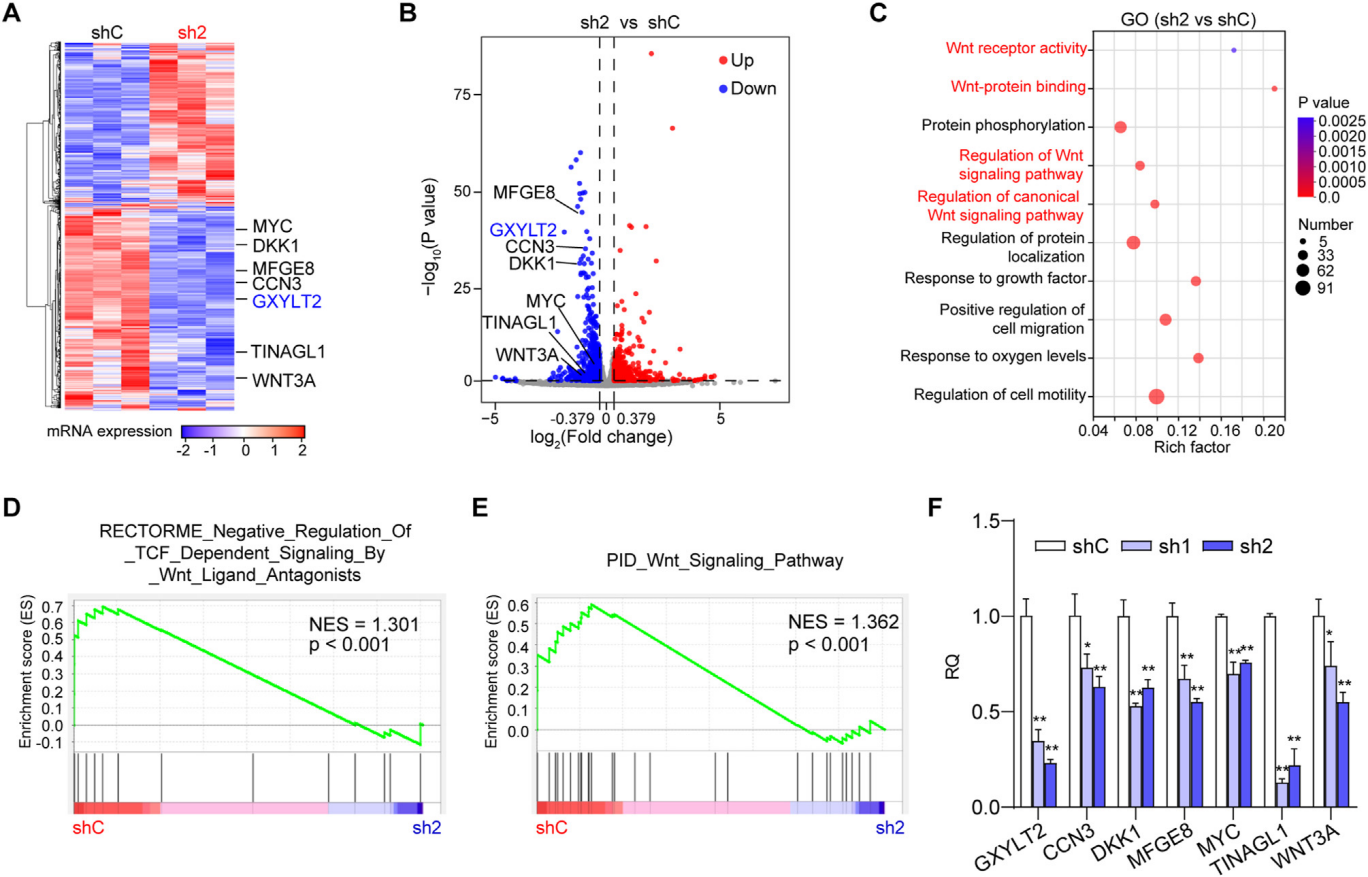
GXYLT2 knockdown repressed the expression of Wnt signaling-related genes in GC cells. **(A)** The heatmap depicting the differentially expressed genes with GXYLT2 knockdown in HGC-27 cells. **(B)** The volcano plot illustrating the differentially expressed genes with GXYLT2 knockdown in HGC-27 cells. Blue dots, down-regulated genes with the fold change < −1.25 and *p* < 0.05; red dots, up-regulated genes with the fold change > 1.25 and *p* < 0.05. **(C)** GO enrichment analysis of the differentially expressed genes with GXYLT2 knockdown in HGC-27 cells. Wnt-related signaling pathways were highlighted in red. **(D**–**E)** GSEA for the correlations between Wnt-related signaling pathways and GXYLT2 knockdown in HGC-27 cells. **(F)** The mRNA levels of Wnt signaling-related genes in HGC-27 cells with GXYLT2 knockdown using quantitative reverse transcription PCR. Data were presented as mean ± standard deviation of three independent experiments. *p*-values were calculated with one-way ANOVA (F). ∗*p* < 0.01 and ∗∗*p* < 0.01. sh2, shGXYLT2; GXYLT2, glucoside xylosyltransferase 2; GC, gastric cancer.

Since GXYLT2 was previously shown to activate Notch1 signaling by up-regulating Hes family bHLH transcription factor 1 (HES1) to facilitate tumor progression,^33^ we investigated whether the suppressive effects of GXYLT2 knockdown on GC aggressiveness were related to Notch1 signaling. Unexpectedly, the protein level of cleaved Notch1 was not remarkably changed in HGC-27 and MKN1 cells with GXYLT2 knockdown (Fig. S7A). RNA sequencing analysis was therefore performed on HGC-27 cells with GXYLT2 knockdown (sh2) and control cells (shC) to explore the downstream targets of GXYLT2 (Fig. 4A). DESeq2 package revealed 1544 genes with significant differences under the criteria of *p* < 0.05 and |fold change| ≥ 1.25 (Fig. 4B). Gene Ontology (GO) enrichment analysis unveiled multiple Wnt-related signaling pathways (Fig. 4C). Moreover, gene set enrichment analysis (GSEA) showed the down-regulation of Wnt-related signaling pathway was positively correlated with GXYLT2 knockdown (NES = 1.301, Fig. 4D; NES = 1.362, Fig. 4E). The components of Wnt signaling pathway with differential expressions were then identified, such as CCN3, DKK1, MFGE8, MYC, TINAGL1, and WNT3A (Fig. 4A, B), and their down-regulation induced by GXYLT2 knockdown was confirmed by quantitative reverse transcription PCR (Fig. 4F). The mRNA expression levels of Notch1 target genes, including HES1, HES2, HES4, HEY2, and HEYL,^34^ were also assessed using the RNA sequencing data. Consistent with the Western blotting data for the cleaved Notch1 level (Fig. S7A), no significant mRNA changes were observed in these Notch1 downstream genes under GXYLT2 knockdown (Fig. S7B). To confirm these findings based on our RNA sequencing data, GSEA was further conducted on two GC public datasets, and identified that a decrease in Wnt-related signaling was positively associated with low expression of GXYLT2 in TCGA-STAD (Fig. S7C, D) and GSE66229 datasets (Fig. S7E, F). Taken together, the potential regulation of GXYLT2 on Wnt signaling, but not the canonical Notch1 signaling, was indicated.

### GXYLT2 knockdown in GC cells decreased the nuclear localization of β-catenin

**Figure 5:**
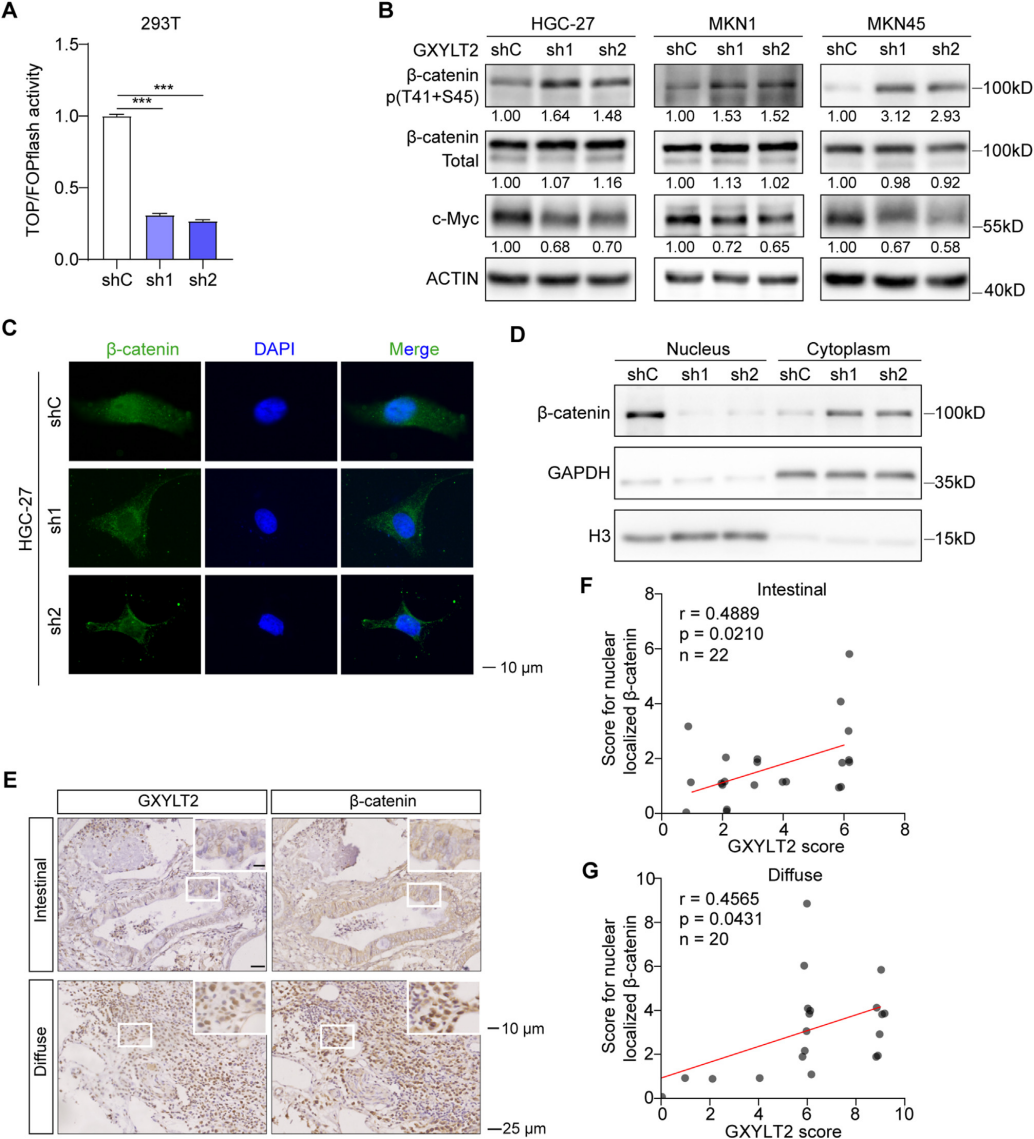
GXYLT2 knockdown suppressed Wnt/β-catenin signaling pathway. **(A)** A TOP/FOPFlash luciferase reporter assay in GXYLT2-knockdown 293T cells. Firefly luciferase activity from TOPFlash or FOPFlash plasmid was normalized by Renilla luciferase activity from pRL-TK plasmid. **(B)** Western blotting analyses for β-catenin phosphorylation and downstream genes of Wnt/β-catenin signaling pathway in HGC-27 (with serum starvation for 48 h), MKN1, and MKN45 cells with GXYLT2 knockdown. **(C)** Immunofluorescence staining for β-catenin localization (green) in GXYLT2-knockdown HGC-27 cells with serum starvation for 48 h. DAPI (blue), nuclei staining. Scale bar, 10 μm. **(D)** The β-catenin expression level in the nucleus and cytoplasm detected by western blotting analysis in HGC-27 (with serum starvation for 48 h). **(E)** Immunohistochemistry staining for GXYLT2 and β-catenin proteins in GC specimens from our own cohort. Representative images of GC samples with intestinal or diffuse subtype were shown. Scale bar, 25 μm; scale bar in inset, 10 μm. **(F, G)** Immunohistochemistry score correlations between GXYLT2 and nuclear localized β-catenin protein in the intestinal (F) and diffuse (G) subtypes from GC samples of our own cohort. Data were presented as mean ± standard deviation of three independent experiments. *p*-values were calculated with one-way ANOVA (A). ∗∗∗*p* < 0.001. GXYLT2, glucoside xylosyltransferase 2; GC, gastric cancer.

Previous studies have highlighted the importance of Wnt/β-catenin signaling in the initiation and progression of diffuse-subtype GC,^35^^,^^36^ so that how GXYLT2 modulated the Wnt/β-catenin signaling pathway was investigated. 293T cells were transfected with TOPFlash plasmid to measure the activation of Wnt/β-catenin signaling. GXYLT2 knockdown led to a significant decrease in luciferase activity compared with the control group (*p* < 0.001; Fig. 5A), indicating that GXYLT2 may regulate the Wnt signaling pathway through β-catenin.

In the absence of Wnt protein, β-catenin is phosphorylated at residues S33, S37, T41, and S45, leading to its degradation and reduced nuclear translocation, thereby suppressing downstream gene expression.^37^^,^ ^38^ Herein, we found the phosphorylated β-catenin (T41 + S45) levels were elevated in three diffuse-subtype GC cells with GXYLT2 knockdown, resulting in the decrease of c-Myc, a well-known downstream target of Wnt/β-catenin signaling (Fig. 5B).^39^ Immunofluorescence staining showed that GXYLT2 knockdown also decreased the nuclear localization of β-catenin in HGC-27 cells (Fig. 5C). Reduced nuclear and increased cytoplasmic β-catenin levels were observed in GXYLT2-knockdown HGC-27 cells (Fig. 5D). Immunohistochemistry staining on human GC tissue sections further showed lower expression of GXYLT2 and decreased nuclear localization of β-catenin in the intestinal-subtype GC samples, compared with the diffuse-subtype ones (Fig. 5E). Immunohistochemistry score for GXYLT2 protein level was positively correlated with the nuclear localized β-catenin protein in both subtypes (Fig. 5F, G).

**Figure 6:**
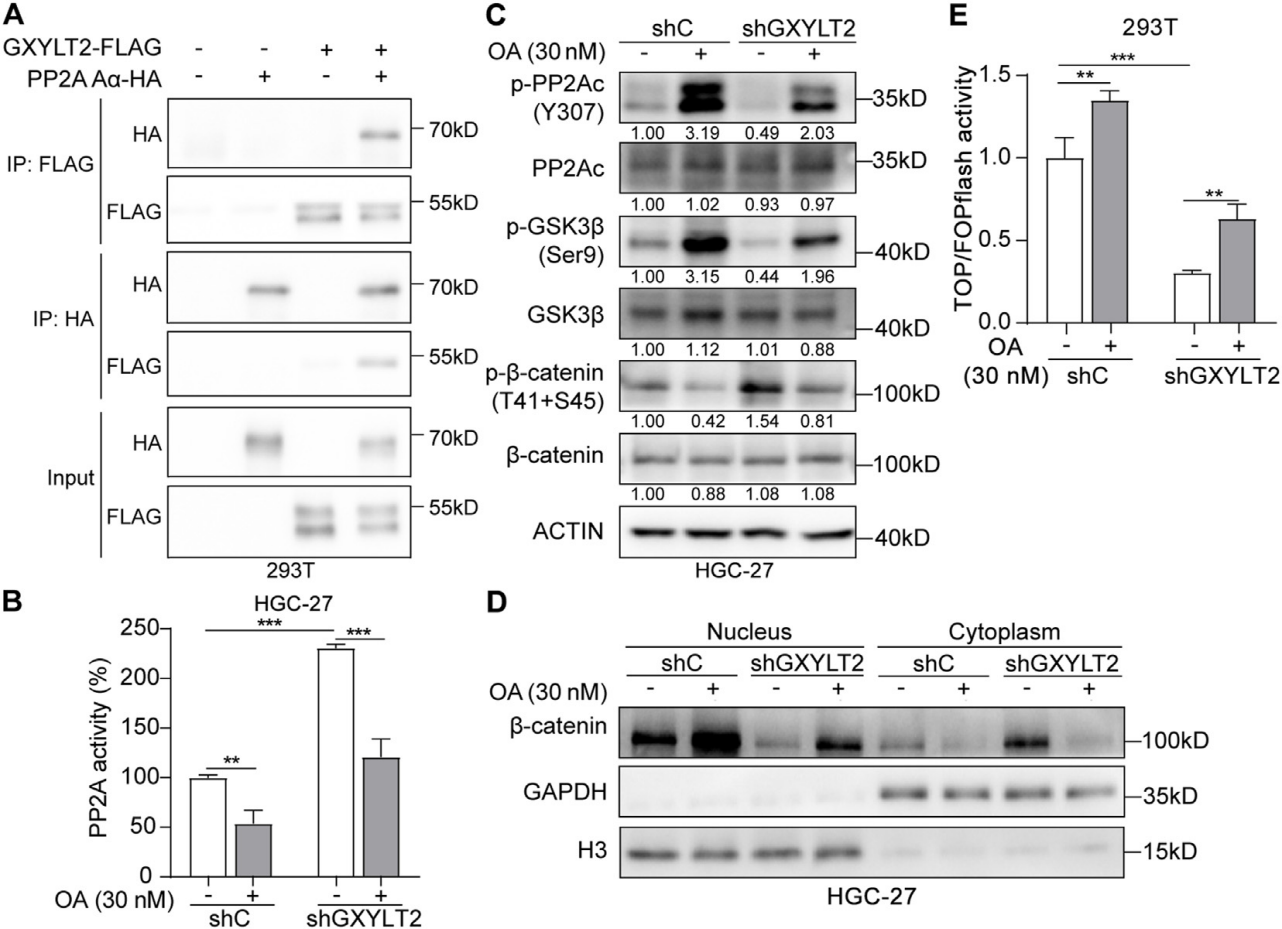
GXYLT2 regulated β-catenin signaling via PP2A complex. **(A)** Co-immunoprecipitation assay in 293T cells transiently transfected with HA-tagged PP2A Aα and FLAG-tagged GXYLT2, using a FLAG-tag antibody for immunoprecipitation and a HA-tag antibody to detect ectopic PP2A Aα (upper panel), and using a HA-tag antibody for immunoprecipitation and a FLAG-tag antibody to detect ectopic GXYLT2 (middle panel). Whole cell lysates were used as the input control (low panel). **(B)** PP2A phosphatase activity in HGC-27 cells with GXYLT2 knockdown (shGXYLT2), with vehicle or okadaic acid (OA, 30 nM) treatment for 8 h shC, mock cells. **(C)** Western blotting analyses for the protein levels of β-catenin signaling in HGC-27 cells (shC or shGXYLT2; with serum starvation for 48 h followed by 8 h of 30 nM OA treatment). **(D)** The β-catenin expression level in the nucleus and cytoplasm detected by western blotting analysis in HGC-27 cells (shC or shGXYLT2; with serum starvation for 48 h followed by 8 h of 30 nM OA treatment). **(E)** TOP/FOPFlash luciferase reporter assay in GXYLT2-knockdown 293T cells with 30 nM OA treatment for 8 h. Firefly luciferase activity from TOPFlash or FOPFlash plasmid was normalized by Renilla luciferase activity from pRL-TK plasmid. Data were presented as mean ± standard deviation of three independent experiments. *p*-values were calculated with two-way ANOVA (B and E). ∗∗*p* < 0.01 and ∗∗∗*p* < 0.001. GXYLT2, glucoside xylosyltransferase 2; PP2A, protein phosphatase 2A.

To investigate whether the role of GXYLT2 in GC was through β-catenin activation, its well-known regulators were examined for the potential interaction with GXYLT2 protein. Protein phosphatase 2A (PP2A) is a critical serine/threonine phosphatase complex, composed of a scaffold subunit (A subunit, PP2A Aα), a regulatory subunit (B subunit), and a catalytic subunit (C subunit, PP2Ac). Previous studies have demonstrated that PP2A could inhibit nuclear translocation of β-catenin via dephosphorylating GSK3β at Ser9.^40^^,^^41^ Thus, the potential protein–protein interaction between PP2A Aα and GXYLT2 was validated by co-immunoprecipitation assay. As shown in Figure 6A, HA-tagged PP2A Aα and FLAG-tagged GXYLT2 were ectopically expressed in 293T cells; HA-tagged PP2A Aα could be co-immunoprecipitated by FLAG antibody, and *vice versa*. Because it was reported that the binding between PP2A Aα and RASAL2 or CFTR could inhibit PP2A activity,^42^^,^^43^ this complex activity was assessed in HGC-27 cells with GXYLT2 knockdown using a malachite green phosphate assay kit. The PP2A inhibitor (okadaic acid) was used as a positive control.^41^ GXYLT2 knockdown increased the PP2A activity (*p* < 0.001), and okadaic acid treatment reversed its effect (*p* < 0.001; Fig. 6B), indicating that GXYLT2 could also inhibit the PP2A activity. Western blotting assays showed that GXYLT2 knockdown decreased the phosphorylation level of PP2Ac, *i.e.*, activated the PP2A complex, resulting in dephosphorylation of GSK3β at Ser9 and sequential phosphorylation of β-catenin (T41 + S45); and okadaic acid treatment could partially restore these effects (Fig. 6C). The β-catenin levels in nucleus (Fig. 6D) and TOPFlash reporter activity (Fig. 6E) confirmed the inhibitory effects of GXYLT2 knockdown on β-catenin translocation and its transcriptional activation activity, which could be counteracted by okadaic acid treatment. How GXYLT2 regulated the β-catenin activation was demonstrated in GC cells.

### GXYLT2 knockdown inhibited the tumorigenicity of GC cells

**Figure 7:**
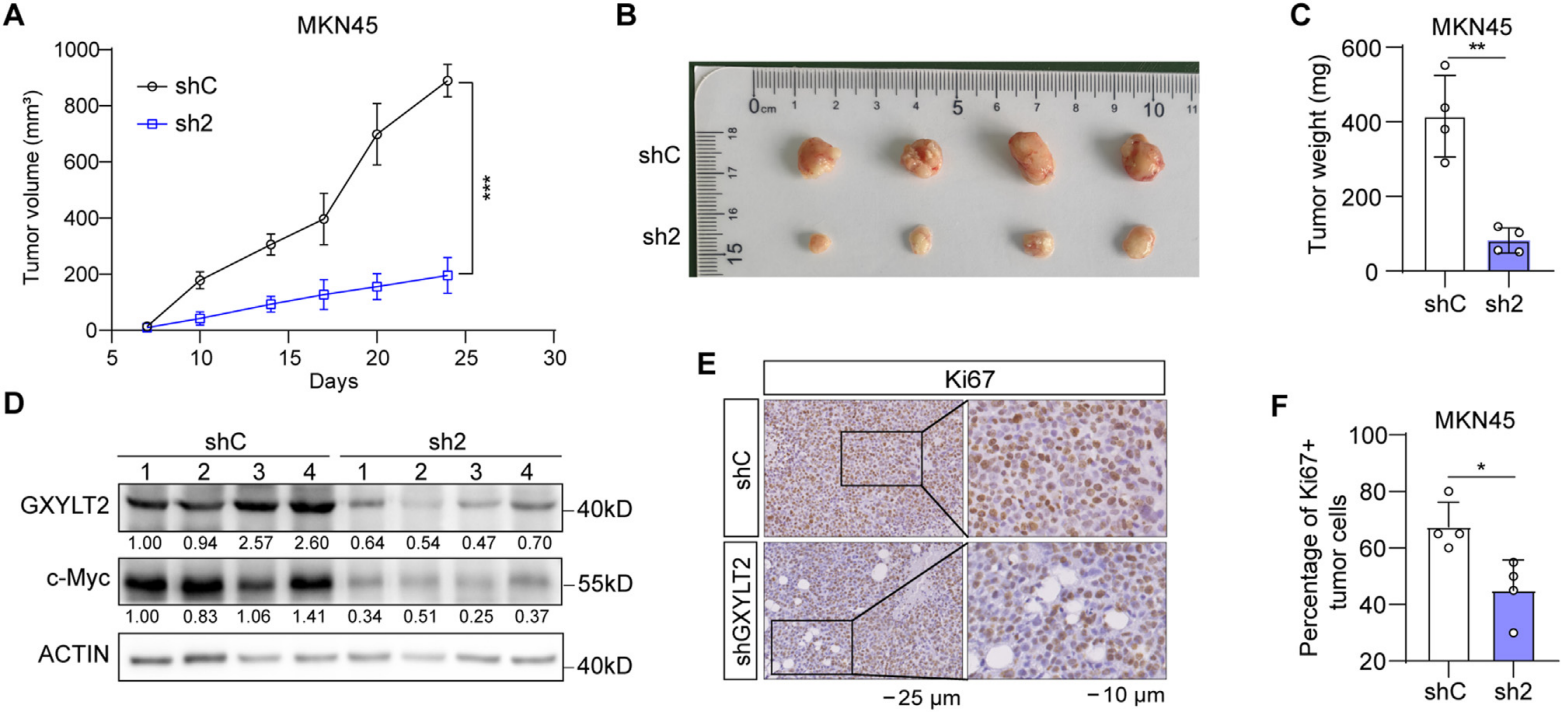
GXYLT2 inhibited the tumorigenicity of GC cells. **(A)** Tumor growth curves of MKN45 cells with GXYLT2 knockdown (sh2) or control (shC) (*n* = 4/group). **(B, C)** Tumor image (B) and weight (C) of MKN45 xenografts (sh2 and shC). **(D)** Western blotting analyses for GXYLT2 and c-Myc protein expression in MKN45 xenografts (sh2 and shC). **(E)** Immunohistochemistry staining for Ki67 expression in MKN45 xenografts (sh2 and shC). Scale bar, 25 μm. **(F)** The percentage of Ki67^+^ cancer cells was quantified in MKN45 xenografts (sh2 and shC). Data were presented as mean ± standard deviation. *p*-values were calculated with one-way ANOVA (A) and two-tailed unpaired student's *t*-test (C and F). ∗*p* < 0.05, ∗∗*p* < 0.01, and ∗∗∗*p* < 0.001. GXYLT2, glucoside xylosyltransferase 2; GC, gastric cancer.

To study the *in vivo* role of GXYLT2, its knockdown in MKN45 cells significantly repressed the tumorigenicity, comparing with the control group (*p* < 0.001; Fig. 7A). At the experimental endpoint, tumors were harvested (Fig. 7B), and tumor weight of control group was remarkably higher than that of GXYLT2 knockdown group (sh2; *p* < 0.01; Fig. 7C). Western blotting analysis confirmed the reduced GXYLT2 protein levels in GXYLT2-knockdown xenografts, accompanied by the decrease of c-Myc protein, a downstream target of the Wnt/β-catenin signaling (Fig. 7D). Additionally, immunohistochemistry staining indicated the reduced proliferation index of GXYLT2-depleted GC xenografts with lower percentage of Ki67^+^ GC cells, compared with the controls (*p* < 0.05; Fig. 7E, F). These findings demonstrated that GXYLT2 knockdown inhibited GC cell proliferation *in vivo*.

## Discussion

Since aberrant glycosylation plays a crucial role in the development and progression of GC, glycosylation-related biomarkers, such as carbohydrate antigen 19-9 (CA19-9), have been successfully utilized in the diagnosis and prognosis of GC.^26^^,^^27^^,^^44^ Currently, the existing methods for classifying GC have obvious limitations. It is necessary and feasible to analyze transcriptome datasets of genes involved in glycosylation, referred to as glycogenes, to guide the molecular classification and uncover novel biomarkers of GC.

Herein, we developed a novel molecular classification based on a 12-glycogene signature enabling the prognosis prediction of GC patients. This 12-glycogene signature-based classification was rigorously juxtaposed against the prevailing classification paradigms, including the Lauren, “Singapore-Duke”, “Mesenchymal”, and ACRG classifications. Across four independent datasets containing 1021 GC patients, we found significant correlations between our classification and the existing ones, *i.e.*, subtypes flagged for an adverse prognosis by conventional classifications were predominantly enriched in cluster I by our 12-glycogene signature approach. Consistently, the overall survival of GC patients in cluster I was significantly worse than that in cluster II. In addition to GXYLT2 in the 12-glycogene signature, glucosaminyl (N-Acetyl) transferase 3 (GCNT3) played a pivotal role in the proliferation and migration of Epstein–Barr virus-associated GC cells; and fucosyltransferase 3 (FUT3), the enzyme for the biosynthesis of all Lewis antigens, was indispensable for GC cell adhesion.^45^^,^^46^ The evidence suggested our 12-glycogene signature as a robust classification criterion for GC patients.

This glycogene-based molecular classification is a powerful approach to identify a gene signature to distinguish poor prognosis based on mRNA level compared with the current molecular stratification.^26^^,^^27^, ^47^ In addition, the GSVA approach can further screen out the most consistent change of one individual glycogene, while in this study, GXYLT2 overexpression in diffuse subtype GC was an excellent example, demonstrating the reliability of this approach. In the future, glycoproteomics approach is expected to fully understand GC development, in particular, combined with multiple omics approaches.

GXYLT2, also known as GLT8D4, is one of the members of the glycosyltransferase gene family 8 in the Carbohydrate-Active EnZymes database.^48^ Different from the enzymatic functions of GLT8D1 and GLT8D2, which are UDP-dependent galactosyltransferases,^49^ GXYLT2, as well as GXYLT1, is in charge of adding the first xylosyl residue from UDP-xylose to *O*-glucose on EGF-like repeats with alpha1,3-linkage.^50^ Notch receptor is a well-documented substrate for GXYLT1 and GXYLT2. The function of xylose modification on the extracellular domain of Notch receptor is context-dependent. The *Drosophila* GXYLT (*Shams*) transferred xylose onto a specific subset of Notch EGF repeats for negative regulation of Notch signaling, while human GXYLT2 activated Notch1 signaling to promote cancer cell proliferation and invasion.^33^ Our study found that GXYLT2 knockdown did not affect the levels of cleaved Notch1 and its downstream target genes, indicating that novel substrates or the glycotransferase activity-independent functions of GXYLT2 should be further explored in GC cells.

Previous researchers have studied GXYLT2 in GC, primarily by comparing the expression level differences between tumor and normal tissues, and they indicated that GXYLT2 played a pro-cancer role in GC.^51^^,^^52^ However, the expression and function of GXYLT2 gene in different subtypes of GC have not been investigated yet. In this study, we revealed the differential expression patterns of GXYLT2 in the intestinal and diffuse subtypes and identified PP2A/GSK3β/β-catenin pathway as a novel downstream signaling of GXYLT2.

**Figure 8:**
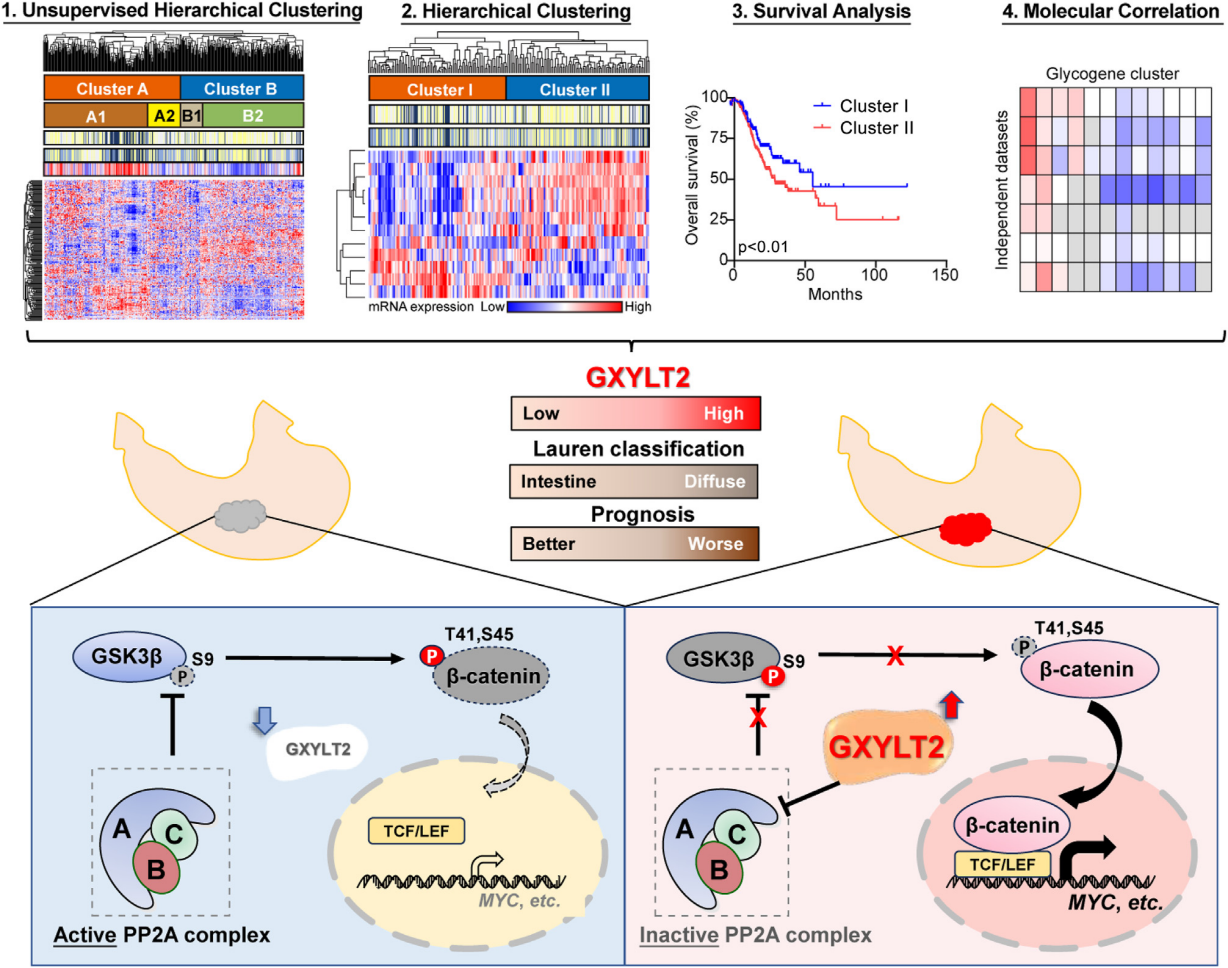
The working model of GXYLT2/β-catenin axis in the diffuse-subtype GC cells, which is associated with glycogene expression and patients' outcomes. GXYLT2, glucoside xylosyltransferase 2; GC, gastric cancer.

Dysregulation of the Wnt signaling pathway plays a significant role in the onset and progression of certain GC,^53^ especially in the advancement of diffuse-subtype GC.^35^^,^^54^^,^^55^ For instance, frequent mutations in *APC* gene led to the stabilization of β-catenin protein in the nucleus and overactivation of Wnt signaling, thereby fostering the progression of GC with diffuse subtype.^36^ We also found that GXYLT2 depletion could only suppress the tumor aggressiveness of the diffuse-subtype GC cells. β-catenin can be regulated by its phosphorylation at S33/S37/T41/S45 to facilitate cytoplasmic β-catenin degradation and reduce its nuclear translocation.^38^^,^^56^ It was reported that *O*-glycosylation of CD44 by GALNT1 led to β-catenin phosphorylation at S675, resulting in the increase of nuclear β-catenin in GC cells.^25^ We demonstrated that GXYLT2 knockdown in the diffuse-subtype GC cells decreased the nuclear localization of β-catenin and repressed the TOPFlash reporter activity, accompanied by the down-regulation of Wnt/β-catenin signaling-downstream genes. Mechanically, GXYLT2 could inhibit PP2A activity via binding to PP2A Aα; and GXYLT2 down-regulation could activate PP2A complex, decrease the phosphorylated GSK3β, increase β-catenin phosphorylation, and prevent β-catenin nuclear translocation from driving the transcription of its downstream targets (Fig. 8). How GXYLT2 regulates Wnt/β-catenin signaling, via glycosylation or not, should be further investigated.

In conclusion**,** this study developed a novel glycogene-based molecular classification strategy and identified GXYLT2 as a potential prognostic biomarker for GC patients. Targeting GXYLT2 suppressed the tumor aggressiveness and inhibited β-catenin activation.

## CRediT authorship contribution statement

**Jiale Yang:** Writing – original draft, Visualization, Validation, Investigation, Data curation, Conceptualization. **Jiajun Wu:** Visualization, Validation, Investigation, Data curation. **Ziqiang Chen:** Visualization, Validation, Investigation, Data curation. **Xiangyun Hou:** Investigation, Data curation. **Xiaojing Li:** Data curation, Investigation. **Zhaorui Liu:** Validation, Investigation, Data curation. **Kai Yin:** Validation, Investigation, Data curation. **Tao Pang:** Writing – review & editing, Resources, Funding acquisition. **Ruimin Huang:** Writing – review & editing, Resources, Funding acquisition, Formal analysis. **Jun Yan:** Writing – review & editing, Resources, Formal analysis, Conceptualization.

## Ethics declaration

Ethical approval was obtained from the Ethics Committee of the First Affiliated Hospital of Naval Medical University (Approval notice: CHEC2024-080). Written informed consent was provided by all participants. All experimental procedures were conducted in accordance with the guidelines and regulations approved by the Institutional Animal Care and Use Committee of the Shanghai Institute of Materia Medica, Chinese Academy of Sciences (Approval notice: 2024-10-HRM-90).

## Funding

This study was supported by the 10.13039/501100001809National Natural Science Foundation of China (No. 82172001 to Ruimin Huang), Shanghai Municipal Science and Technology Major Project (China) (to Ruimin Huang), and the Navy Medical University Foundation (China) (No. 2021MS07 to Tao Pang).

## Conflict of interests

The authors declared no competing interests.
