# Supplementary material for: GXYLT2 serves as a prognostic biomarker and is associated with β-catenin activation and gastric cancer aggressiveness
Source: Genes Dis. 2025 May 5;13(2):101673. doi: 10.1016/j.gendis.2025.101673 (PMC12765262; doi:10.1016/j.gendis.2025.101673)
Supplement: Multimedia component 1 [file mmc1.docx]

**Supporting Information**

The seven supplementary figures and nine supplementary tables referenced in the main document can be found in Doc S1. A summary of the *Materials and Methods* is provided in the main document.


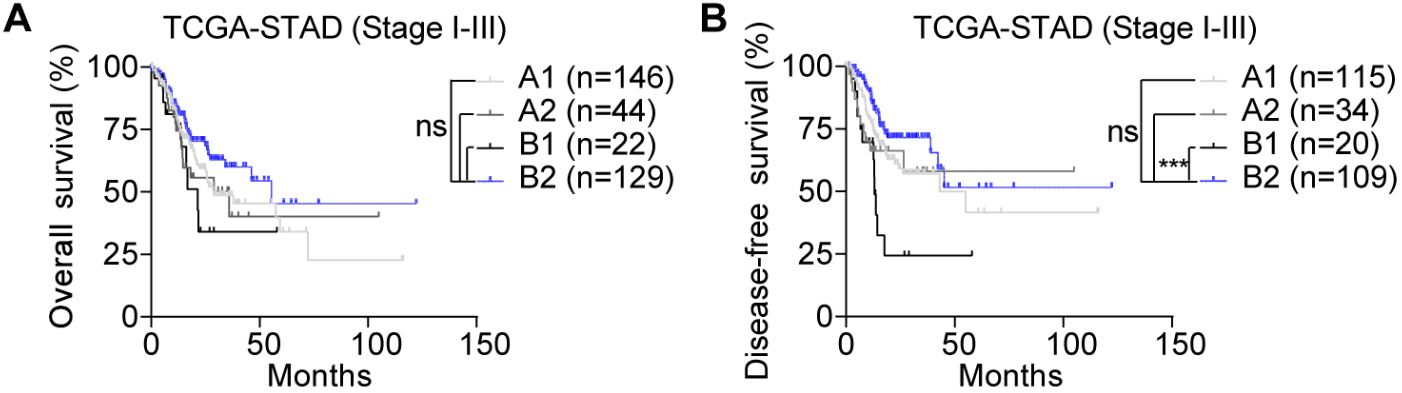


**Figure S1** Kaplan-Meier plots for overall survival (**A**) and disease-free survival (**B**) in GC patients at stage I-III from TCGA-STAD dataset, comparing that in subcluster B2 with other three subclusters (A1, A2 and B1) individually. *p* values were assessed by log-rank test. *** *p* < 0.001; ns, *p* ≥ 0.05.


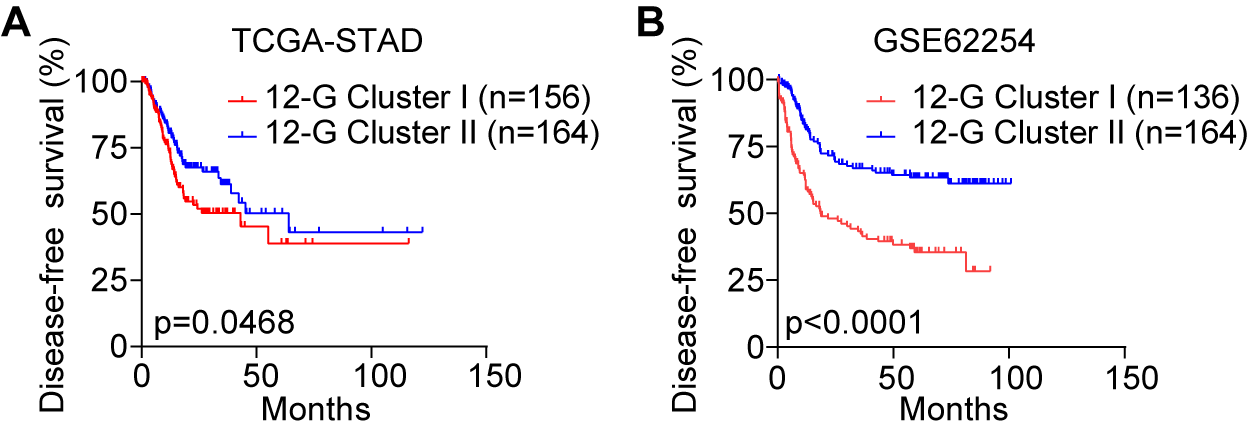


**Figure S2** Kaplan-Meier plots for disease-free survival in GC patients from TCGA-STAD (**A**) and GSE62254 (**B**) datasets, stratified by 12-glycogene signature classification as Cluster I and II. *p* values were assessed by log-rank test.


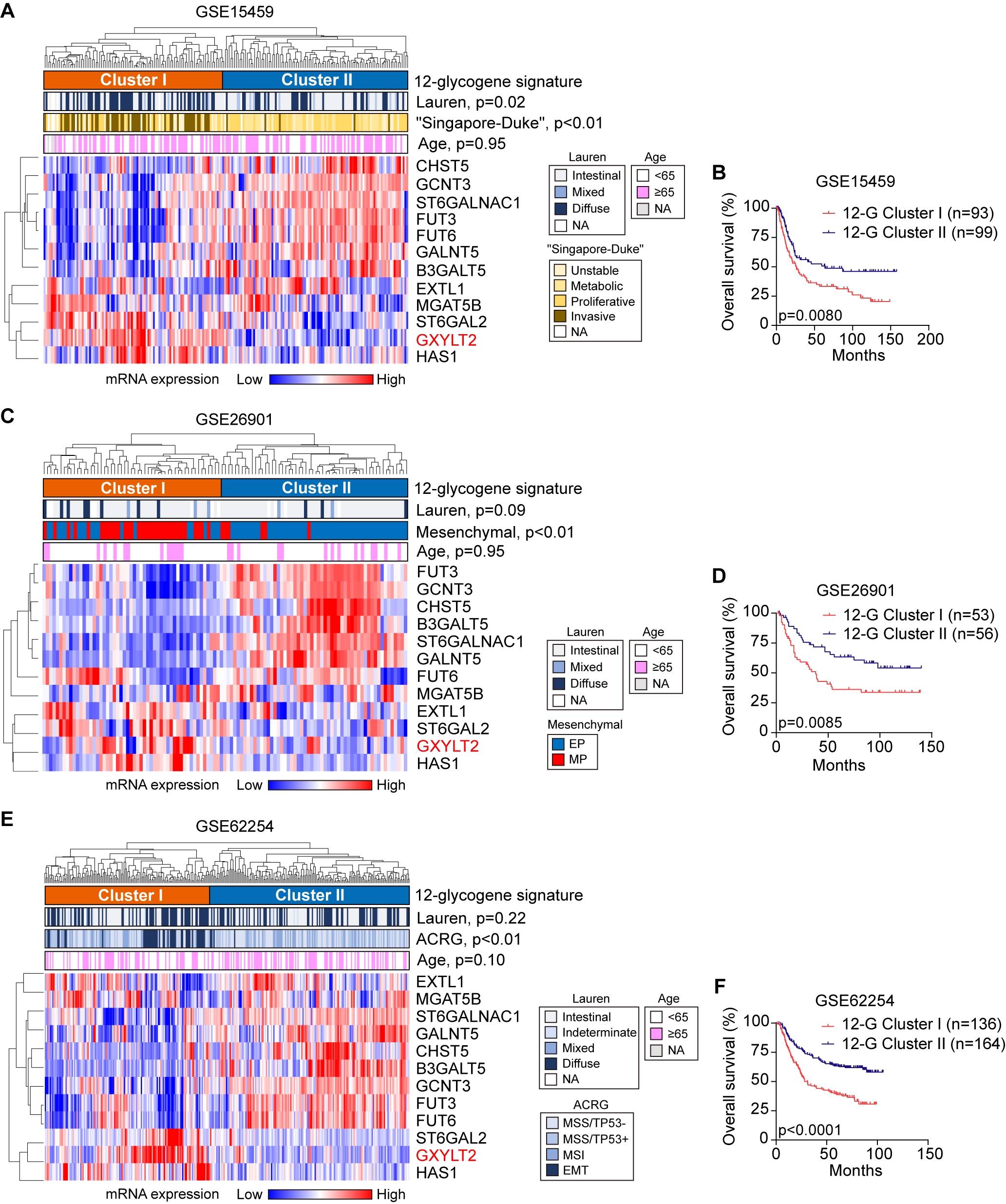


Figure S3 Identification of a molecular classification based on 12-glycogene signature in gastric cancer patients. (A) Clustering based on 12-glycogene signature in GC patients from GSE15459 dataset. Two clusters (I and II) were compared with the Lauren classification (Intestinal, Diffuse, and Mixed), “Singapore-Duke” classification (Unstable, Metabolic, Proliferative, and Invasive), and age, respectively. (B) Kaplan-Meier plot for overall survival in GC patients from GSE15459 dataset, stratified by Cluster I and II. (C) Clustering based on 12-glycogene signature in GC patients from GSE26901 dataset. Two clusters (I and II) were compared with the Lauren classification, the “Mesenchymal” classification (EP and MP), and age, respectively. (D) Kaplan-Meier plot for overall survival in GC patients from GSE26901 dataset, stratified by Cluster I and II. (E) Clustering based on 12-glycogene signature in GC patients from GSE62254 dataset. Two clusters (I and II) were compared with the Lauren classification, the ACRG classification (MSS/TP53^-^, MSS/TP53^+^, MSI, and EMT), and age, respectively. (F) Kaplan-Meier plot for overall survival in GC patients from GSE62254 dataset, stratified by Cluster I and II. *p* values were calculated with 𝜒^2^ test (A, C and E) and with log-rank test (B, D and F).


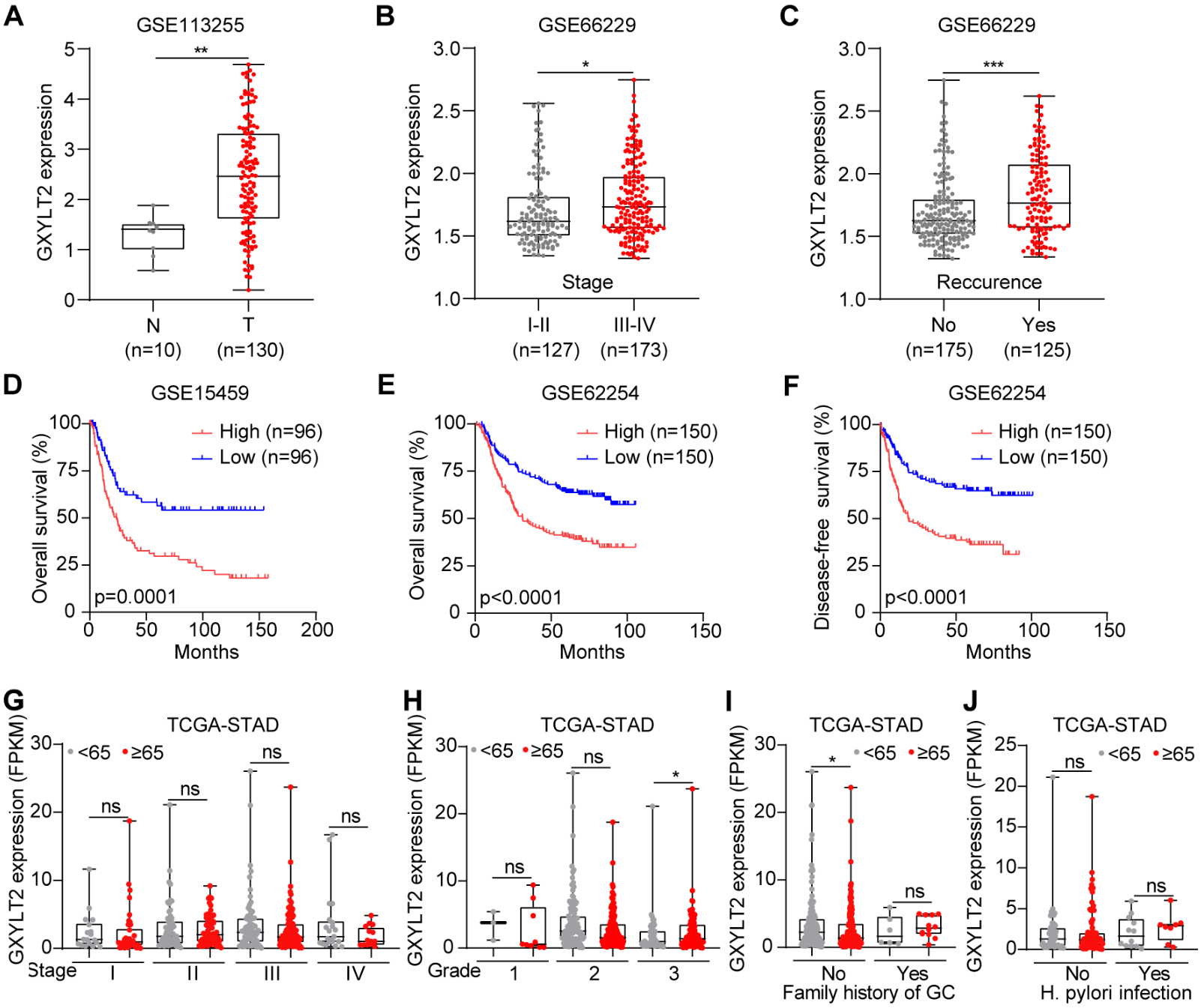


**Figure S4** The correlation between GXYLT2 expression and clinical characteristics in GC patients. (**A**) GXYLT2 mRNA levels in GC and normal gastric tissues from GSE113255 dataset. (**B**) Correlation between GXYLT2 mRNA levels and tumor stage of GC patients in GES66229 dataset. (**C**) The association of GXYLT2 mRNA levels with recurrence status in GES66229 dataset. (**D, E**) Kaplan-Meier analysis of overall survival of GC patients from GSE15459 (D) and GSE62254 datasets (E). (**F**) Kaplan-Meier analysis of disease-free survival of GC patients in GSE62254 dataset. Data were presented as means ± SD. **(G–J)** The correlations between GXYLT2 mRNA levels and clinical characteristics in GC patients with different tumor stages (I-IV; G), tumor grades (1-3; H), family history of GC (I), and H. pylori infection (J), stratified by patients’ age as < 65 and ≥ 65-year old, in TCGA-STAD dataset. *p* values were calculated with two-tailed unpaired student’s *t*-test (A-C & G-J**)** and with log-rank test (D–F). ^*^*p* < 0.05; ^**^*p* < 0.01; ^***^*p* < 0.001; ns, *p* ≥ 0.05.


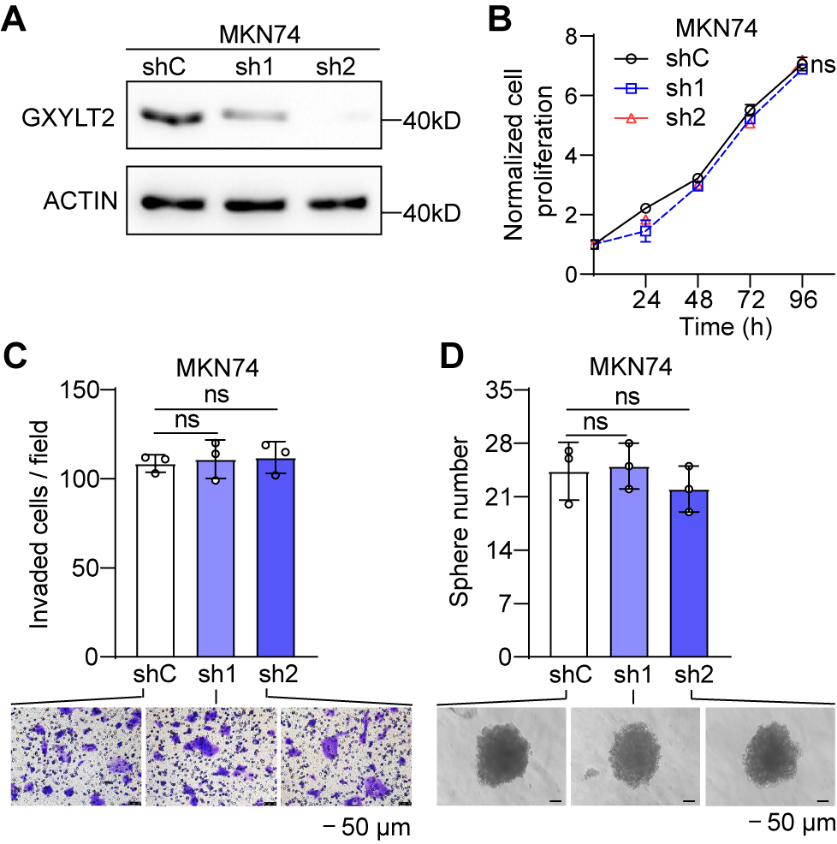


**Figure S5** GXYLT2 knockdown had no significant effects on the proliferation, invasion and sphere formation of intestinal-subtype GC cells. (**A**) GXYLT2 knockdown by shRNAs (sh1 or sh2) in MKN74 cells, detected by Western blotting analysis. shC, control cells. (**B**) The proliferation of GXYLT2-knockdown MKN74 cells by SRB assay. (**C**) The invasion of GXYLT2-knockdown MKN74 cells by transwell assay. (**D**) Sphere formation ability of GXYLT2-knockdown MKN74 cells. Scale bar, 50 μm. Data were presented as means ± SD of three independent experiments. *p* values were calculated with two-way ANOVA (B) and one-way ANOVA (C and D). ns, *p* ≥ 0.05.


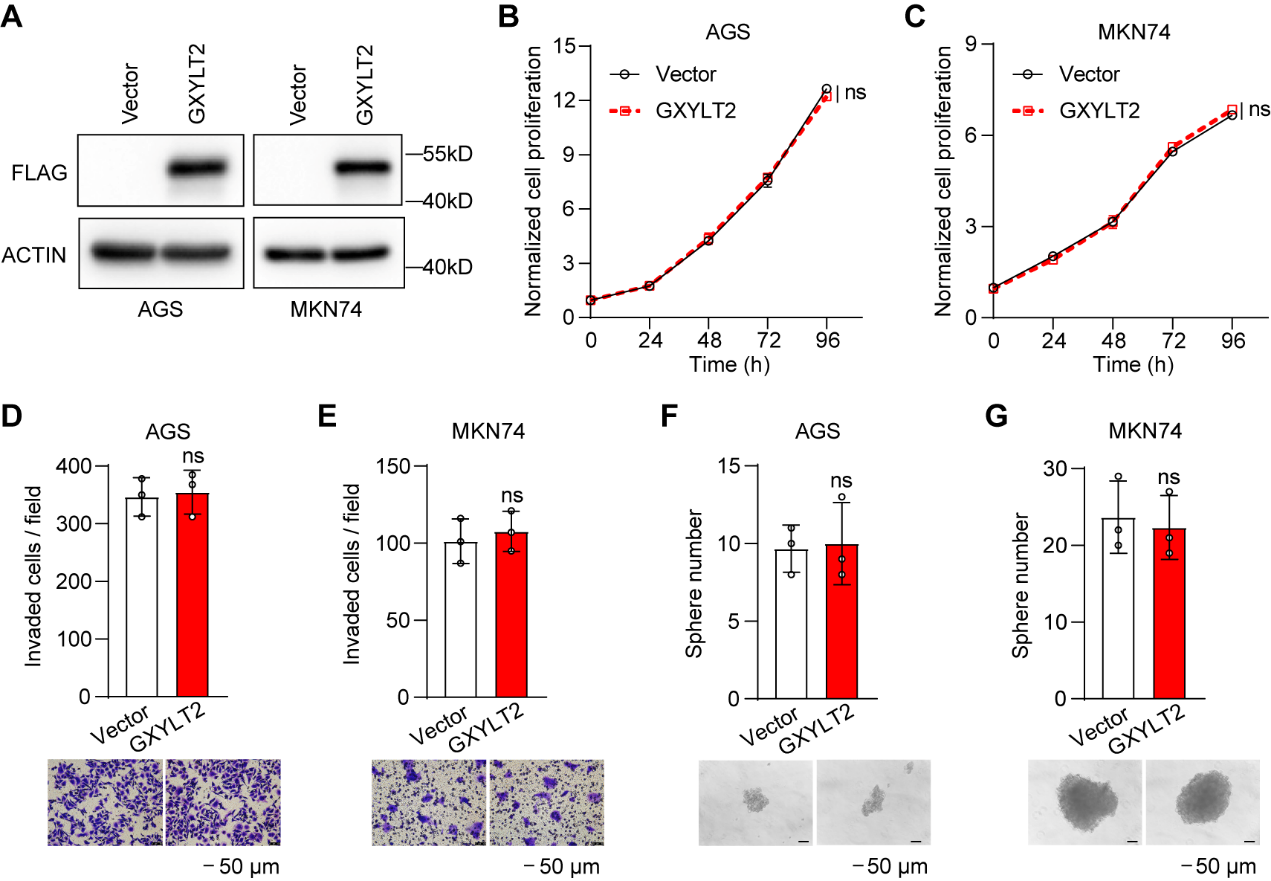


**Figure S6** GXYLT2 overexpression had no significant effects on the proliferation, invasion and sphere formation of intestinal-subtype GC cells. (**A**) Ectopic overexpression of GXYLT2 in AGS and MKN74 cells, detected by Western blotting analysis. Vector, control cells. (**B, C**) The proliferation of GXYLT2-overexpressed AGS (B) and MKN74 (C) cells by SRB assay. (**D, E**) The invasion of GXYLT2-overexpressed AGS (D) and MKN74 (E) cells by transwell assay. (**F, G**) Sphere formation ability of GXYLT2-overexpressed AGS (F) and MKN74 (G) cells. Scale bar, 50 μm. Data were presented as means ± SD of three independent experiments. *p* values were calculated with one-way ANOVA (B and C) and two-tailed unpaired Student’s *t*-test (D–G). ns, *p* ≥ 0.05.


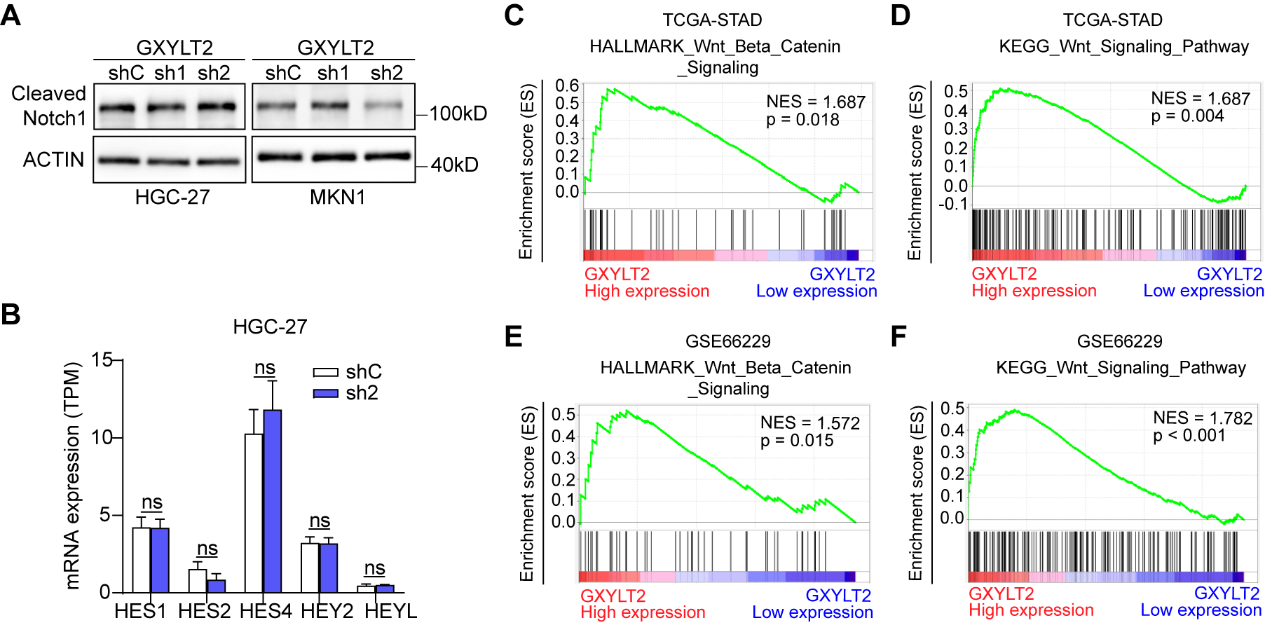


**Figure S7** GXYLT2 knockdown repressed the expressions of Wnt signaling-related genes in GC cells. (**A**) Western blotting analysis for the cleaved Notch1 protein in HGC-27 and MKN1 cells with GXYLT2 knockdown (sh1 or sh2). shC, control cells. (**B**) The mRNA levels of Notch1 signaling-related genes in HGC-27 cells with GXYLT2 knockdown using RNA-seq data. (**C–F**) GSEA for the correlations between Wnt-related signaling pathways and GXYLT2 expression in TCGA-STAD (C, D) and GSE66229 datasets (E, F). Data were presented as means ± SD of three independent experiments. *p* values were calculated with two-tailed unpaired Student’s *t*-test (B). ns, *p* ≥ 0.05. sh2: shGXYLT2.

Table S1 Antibodies for Western blotting, immunochemistry, immunofluorescence staining, and co-immunoprecipitation assays.

| Antibody | Company | Cat # | RRID | Application (Dilution) |
| --- | --- | --- | --- | --- |
| Actin | Sigma | A2228 | AB_476697 | WB (1:2,500) |
| total β-catenin | Abways | CY3523 | NA | WB (1:1,000) |
| β-catenin | Proteintech | 66379-1-Ig | NA | IF (1:200)  IHC (1:500) |
| Phospho-(T41+S45) β-catenin | Abways | CY9969 | NA | WB (1:1,000) |
| Flag | Sigma | F1804 | AB_262044 | WB (1:2,500) |
| GAPDH | Proteintech | 60004-1-Ig | AB_2737588 | WB (1:10,000) |
| p-GSK3β (S9) | HUABIO | ET1607-60 | AB_3069776 | WB (1:1000) |
| GSK 3β | Proteintech | 22104-1-AP | AB_2878997 | WB (1:500) |
| GXYLT2 | Absin | abs103978 | NA | WB (1:1,000)  IHC (1:200) |
| HA | CST | 3724 | AB_1549585 | WB (1:2000) |
| Histone H3 | CST | 9715 | AB_331563 | WB (1:2,000) |
| Ki67 | Abcam | Ab15580 | AB_443209 | IHC (1:400) |
| c-Myc | CST | 13978 | AB_2631168 | WB (1:1,000) |
| cleaved NOTCH1 | CST | 2421 | AB_2314204 | WB (1:1,000) |
| p-PP2AC(Y307) | HUABIO | ET1609-40 | AB_3069850 | WB (1:500) |
| PP2AC | Proteintech | 13482-1-AP | AB_2169485 | WB (1:500) |
| goat anti-mouse IgG secondary antibody, 488 nm | Invitrogen | A32732 | NA | IF (1:500) |
| goat α-rabbit HRP  2^nd^ antibody | Jackson ImmunoResearch | 846738 | NA | WB (1:2,500) |
| goat α-mouse HRP 2^nd^ antibody | Jackson ImmunoResearch | 847273 | NA | WB (1:2,500) |

**Notes**: CST: Cell Signaling Technology; WB, Western blotting; IHC, immunohistochemistry; IF, immunofluorescence.

Table S2 The sequences for PCR primers and shRNAs.

| **Name** | **Direction** | **Sequences (5’-3’)** |
| --- | --- | --- |
| **qRT-PCR primers** | | |
| ACTB | Forward | CATGTACGTTGCTATCCAGGC |
|  | Reverse | CTCCTTAATGTCACGCACGAT |
| CCN3 | Forward | CACGGCGGTAGAGGGAGATAA |
|  | Reverse | TGGGCCACAGATCCACTTTTC |
| DKK1 | Forward | ATAGCACCTTGGATGGGTATTCC |
|  | Reverse | CTGATGACCGGAGACAAACAG |
| GXYLT2 | Forward | TCTGAAGCCCGAGTTTGATAAGC |
|  | Reverse | TGATGGGGTAGATTCTGTGCT |
| MFGE8 | Forward | CCTGCCACAACGGTGGTTTAT |
|  | Reverse | CACATTTCGTCTCACAGTGGTT |
| MYC | Forward | GGCTCCTGGCAAAAGGTCA |
|  | Reverse | CTGCGTAGTTGTGCTGATGT |
| TINAGL1 | Forward | ACCAGGTCACTCCTGTCTACC |
|  | Reverse | TGCCTCCCTTGTATAGGAAGAA |
| WNT3A | Forward | ATGAACCGCCACAACAACGAGG |
|  | Reverse | GTCCTTGAGGAAGTCACCGATG |
| **shRNA sequences** | | |
| shGXYLT2 | sh1 | CCGGAGACGCTGGTCATGCTCAAATCTCGAGATTTGAGCATGACCAGCGTCTTTTTTG |
|  | sh2 | CCGGCCAGTGGCCTGACTCATATACCTCGAGGTATATGAGTCAGGCCACTGGTTTTTG |

Table S3 List for kits and reagents.

| **Kits and Reagents** | **Company** | **Cat #** |
| --- | --- | --- |
| BCA protein assay kit | Beyotime | P0011 |
| BeyoECL Star | Beyotime | P0018AM |
| ChamQ SYBR qPCR Master Mix | Vazyme | Q711 |
| Crystal violet | China National Pharmaceutical Group | 71012314 |
| DAB detection kit | MXB Biotechnologies | DAB-2031 |
| DMSO | Meilunbio | PWL064 |
| Dual luciferase reporter gene assay kit | Beyotime | RG027 |
| DMEM | Corning | 10-013-CV |
| EGF, human recombinant protein | PeproTech | GMP100-15 |
| Fetal bovine serum (FBS) | Gibco | 10091148 |
| bFGF, human recombinant protein | PeproTech | AF-100-18B |
| 4% Formaldehyde fixative solution | Beyotime | P0099 |
| Growth factor-reduced Matrigel | Corning | 354234 |
| Hifair II 1^st^ strand cDNA synthesis SuperMix for qPCR (gDNA digester plus) | YEASEN | 11123ES60 |
| Hoechst | Thermo Scientific | 33342 |
| Malachite green phosphate assay kit | Sigma | MAK307 |
| NP40 | Beyotime | P0013F |
| Nitrocellulose membranes (0.45 μm) | Amersham | GE10600003 |
| Nuclear and Cytoplasmic Protein Extraction Kit | Beyotime | P0028 |
| Okadaic acid (OA) | Yuanye Biotechnology | S30686 |
| Phosphatase Inhibitor Cocktail Tablets | Roche | 4906845001 |
| Phenylmethanesulfonyl fluoride (PMSF) | Beyotime | ST506 |
| Polybrene | Sigma-Aldrich | H9268 |
| Polyethylenimine (PEI) | Sigma-Aldrich | 408727 |
|  | | |
| **Table S3** List for kits and reagents (Cont’d) | | |
| **Kits and Reagents** | **Company** | **Cat #** |
| Protein A/G PLUS-Agarose | Santa Cruz | sc-2003 |
| Puromycin | Selleck | 58-58-2 |
| Protease Inhibitor Cocktail Tablets | Roche | 4693116001 |
| RPMI 1640 | Corning | 10-040-CV |
| Sodium Dodecyl Sulfate (SDS) | YEASEN | 20106ES76 |
| Sulforhodamine B | Meilunbio | MB1808 |
| Triton X-100 | Sigma | T87870 |
| Trizol reagent | Invitrogen | 15596026 |
| Transwell insert | BIOFIL | TCS-003-024 |
| Ultra-low attachment 6-well plates | Corning | 3417 |

Table S4 The 12 glycogenes differentiated subcluster B2 from the other three subclusters in the TCGA-STAD dataset.

| **Gene Symbol** | **ID** | **Description** | **Log2FC** | **B2 vs Others *p* value** |
| --- | --- | --- | --- | --- |
| *B3GALT5* | 10317 | beta-1,3-galactosyltransferase 5 | -1.1567 | < 0.0001 |
| *CHST5* | 23563 | carbohydrate sulfotransferase 5 | -1.6010 | < 0.0001 |
| *EXTL1* | 2134 | exostosin like glycosyltransferase 1 | 1.7342 | < 0.0001 |
| *FUT3* | 2525 | fucosyltransferase 3 | -1.5370 | < 0.0001 |
| *FUT6* | 2528 | fucosyltransferase 6 | -1.0568 | 0.0095 |
| *GXYLT2* | 727936 | glucoside xylosyltransferase 2 | 1.4981 | < 0.0001 |
| *GCNT3* | 9245 | glucosaminyl (N-acetyl) transferase 3 | -1.4159 | < 0.0001 |
| *HAS1* | 3036 | hyaluronan synthase 1 | 1.4261 | < 0.0001 |
| *MGAT5B* | 146664 | beta-N-acetylglucosaminyltransferase B | 1.6921 | < 0.0001 |
| *ST6GAL2* | 84620 | ST6 beta-galactoside alpha-2,6-sialyltransferase 2 | 1.6144 | < 0.0001 |
| *ST6GALNAC1* | 55808 | ST6 N-acetylgalactosaminide alpha-2,6-sialyltransferase 1 | -1.4896 | < 0.0001 |

**Notes**: “Others” included A1, A2, and B1 subtypes; Log_2_FC, Log_2_(Fold change).

Table S5 Univariate and multivariate analyses of 12-glycogene signature for clinical outcomes in TCGA-STAD dataset.

| Variables | Univariate analysis | | Multivariate analysis | |
| --- | --- | --- | --- | --- |
|  | **HR (95% CI)** | ***p* value** | **HR (95% CI)** | ***p* value** |
| Overall survival (OS) | | | | |
| Cluster  (I *vs* II) | 0.645 (0.469-0.888) | **0.0071** | 0.632(0.449 - 0.889) | **0.0084** |
| Age (Continuous) | 1.022 (1.006-1.037) | **0.0056** | 1.032(1.015 - 1.049) | **<0.0001** |
| Gender (Female vs Male) | 1.218 (0.871-1.704) | 0.2497 | 1.234(0.867 - 1.755) | 0.2426 |
| Grade (1-2 *vs* 3) | 1.459 (1.043-2.040) | **0.0272** | 1.375(0.960 - 1.969) | 0.0822 |
| Stage  (I-II *vs* III-IV) | 2.002 (1.419-2.825) | **<0.0001** | 1.974(1.387 - 2.815) | **<0.0001** |
| Disease-free survival (DFS) | | | | |
| Cluster  (I *vs* II) | 0.680 (0.464-0.997) | **0.0481** | 0.623(0.416 - 0.931) | **0.0209** |
| Age (Continuous) | 0.999 (0.982-1.016) | 0.8775 | 1.006(0.989 - 1.025) | 0.4805 |
| Gender (Female *vs* Male) | 1.928 (1.223-3.039) | **0.0047** | 2.088(1.304 - 3.341) | **0.0022** |
| Grade (1-2 *vs* 3) | 1.361 (0.914-2.029) | 0.1297 | 1.245(0.824 - 1.882) | 0.2973 |
| Stage  (I-II *vs* III-IV) | 1.582 (1.068-2.345) | **0.0223** | 1.441(0.967 - 2.147) | **0.0725** |

**Notes**: HR, Hazard ratio; CI, Confidence interval.

Table S6 Univariate and multivariate analyses of 12-glycogene signature for clinical outcomes in the GSE15459 dataset.

| Variables | Univariate analysis | | Multivariate analysis | |
| --- | --- | --- | --- | --- |
|  | **HR (95% CI)** | ***p* value** | **HR (95% CI)** | ***p* value** |
| Cluster  (I *vs* II) | 0.576 (0.381-0.871) | **0.0088** | 0.602 (0.397-0.912) | **0.0168** |
| Age (Continuous) | 1.000 (0.984-1.016) | 0.9679 | 1.006 (0.991-1.021) | 0.4615 |
| Gender (Female *vs* Male) | 1.402 (0.908-2.165) | 0.1271 | 0.953 (0.609-1.492) | 0.8331 |
| Stage  (1-2 *vs* 3-4) | 6.521 (3.598-11.823) | **<0.0001** | 6.596 (3.594-12.108) | **<0.0001** |

**Notes**: HR, Hazard ratio; CI, Confidence interval.

Table S7 Univariate and multivariate analyses of 12-glycogene signature for clinical outcomes in the GSE26901 dataset.

| Variables | Univariate analysis | | Multivariate analysis | |
| --- | --- | --- | --- | --- |
|  | **HR (95% CI)** | ***p* value** | **HR (95% CI)** | ***p* value** |
| Overall survival (OS) | | | | |
| Cluster  (I *vs* II) | 0.490 (0.285-0.842) | **0.0098** | 0.441 (0.252-0.770) | **0.0040** |
| Age (Continuous) | 1.043 (1.013-1.074) | **0.0046** | 1.038 (1.007-1.069) | **0.0159** |
| Gender  (Female *vs* Male) | 1.391 (0.784-2.468) | 0.2587 | 1.771 (0.976 - 3.215) | 0.0602 |
| Stage  (I-II *vs* III-IV) | 4.285 (2.378-7.721) | **<0.0001** | 4.141 (2.276 - 7.533) | **<0.0001** |
| Recurrence-free survival (RFS) | | | | |
| Cluster  (I *vs* II) | 0.610 (0.366-1.015) | **0.0571** | 0.552 (0.326-0.935) | **0.0271** |
| Age (Continuous) | 1.052 (1.023-1.083) | **0.0005** | 1.046 (1.016-1.077) | **0.0026** |
| Gender  (Female *vs* Male) | 1.342 (0.778-2.316) | 0.2908 | 1.499 (0.855-2.627) | 0.1573 |
| Stage  (I-II *vs* III-IV) | 3.526 (2.048-6.069) | **<0.0001** | 3.171 (1.831-5.493) | **<0.0001** |

Notes: HR, Hazard ratio; CI, Confidence interval.

Table S8 Univariate and multivariate analyses of 12-glycogene signature for clinical outcomes in the GSE62254 dataset.

| Variables | Univariate analysis | | Multivariate analysis | |
| --- | --- | --- | --- | --- |
|  | **HR (95% CI)** | ***p* value** | **HR (95% CI)** | ***p* value** |
| Overall Survival (OS) | | | | |
| Cluster  (I *vs* II) | 0.461 (0.333-0.637) | **<0.0001** | 0.494 (0.356-0.686) | **<0.0001** |
| Age (Continuous) | 1.011 (0.995-1.026) | 0.1812 | 1.024 (1.007-1.040) | **0.0041** |
| Gender  (Female *vs* Male) | 0.905 (0.647-1.265) | 0.5587 | 0.869 (0.620-1.217) | 0.4144 |
| Stage  (I-II *vs* III-IV) | 3.472 (2.385-5.053) | **<0.0001** | 3.504 (2.393-5.131) | **<0.0001** |
| Disease-free survival (DFS) | | | | |
| Cluster  (I *vs* II) | 0.420 (0.294-0.601) | **<0.0001** | 0.461 (0.321-0.662) | **<0.0001** |
| Age (Continuous) | 1.003 (0.987-1.021) | 0.6762 | 1.015 (0.998-1.032) | 0.0935 |
| Gender  (Female *vs* Male) | 0.967 (0.669-1.402) | 0.8602 | 1.011 (0.696-1.469) | 0.9555 |
| Stage  (I-II *vs* III-IV) | 4.163 (2.681-6.466) | **<0.0001** | 4.026 (2.580-6.283) | **<0.0001** |

Notes: HR, Hazard ratio; CI, Confidence interval.

Table S9 Associations between GXYLT2 expression and clinic-pathological parameters in 42 gastric cancer samples from our cohort.

| **Characteristics** | **n** | **Expression of GXYLT2** | | ***p* value** |
| --- | --- | --- | --- | --- |
|  |  | **High (n, %)** | **Low (n, %)** |  |
| **Gender** |  |  |  | 0.4631 |
| Male | 26 | 16 (38.10%) | 10 (23.81%) |  |
| Female | 16 | 8 (19.05%) | 8 (19.05%) |  |
| **Age** |  |  |  | 0.1084 |
| < 65 | 20 | 14 (33.33%) | 6 (14.29%) |  |
| ≥ 65 | 22 | 10 (23.81%) | 12 (28.57%) |  |
| **T stage** |  |  |  | 0.7262 |
| T1 | 6 | 3 (7.14%) | 3 (7.14%) |  |
| T2 | 7 | 3 (7.14%) | 4 (9.52%) |  |
| T3 | 25 | 15 (35.71%) | 10 (23.81%) |  |
| T4 | 4 | 3 (7.14%) | 1 (2.38%) |  |
| **N stage** |  |  |  | **0.0270** |
| N0 | 11 | 4 (9.52%) | 7 (16.67%) |  |
| N1 | 5 | 5 (11.90%) | 0 (0%) |  |
| N2 | 9 | 8 (19.05%) | 1 (2.38%) |  |
| N3 | 7 | 4 (9.52%) | 3 (7.14%) |  |
| NA | 10 | 3 (7.14%) | 7 (16.67%) |  |
| **Lauren classification** |  |  |  | **0.0043** |
| Intestinal | 22 | 8 (19.05%) | 14 (33.33%) |  |
| Diffuse | 20 | 16 (38.10%) | 4 (9.52%) |  |

Supplementary Materials and Methods

Western blotting assay

Total protein was extracted using 2% SDS lysis buffer supplemented with EDTA-free Protease (#4693132001; Merck) and Phosphatase Inhibitor Cocktail (#4906845001; Merck), were boiled in SDS loading buffer. 10-30 μg protein lysates were loaded to SDS-PAGE and transferred to 0.45 μm nitrocellulose membranes (GE10600003; Amersham). After the membranes were blocked by 5% non-fatty milk or BSA for 60 min, the incubation of primary antibodies, including anti-GXYLT2 (1:1,000, abs103978; Absin), anti-FLAG (1:2,500, F1804-1; Sigma), anti-phospho-(T41+S45) β-catenin (1:1,000, CY9969; Abways), anti-Total β-catenin (1:1,000, CY3523; Abways), anti-c-Myc (1:1,000, #13987; Cell Signaling Technology (CST)), anti-Cleaved Notch1 (Val1744) (1:1,000, #4147; CST), anti-Histone H3 (1:2,000, #9715; CST), anti-HA (1:2,000, #3724; CST), anti-phospho-(S9) GSK3β (1:1,000, ET1607-60; HUABIO), anti-GSK3β (1:500, 22104-1-AP; Proteintech), anti-phospho-(Y307) PP2Ac (1:500, ET1609-40; HUABIO), anti-PP2Ac (1:500, 13482-1-AP; Proteintech), anti-GAPDH (1:10,000, 60004-1-Ig; Proteintech), and anti-β-Actin (1:2,500, A2228; Sigma), was respectively performed at 4℃ overnight. After incubation with corresponding secondary antibodies, the blots were detected with SuperSignal West Pico PLUS chemiluminescent substrate (#34580; Thermo Fisher Scientific). Antibody information was listed in Table S1.

IHC staining

Formalin-fixed paraffin-embedded tissues were sectioned into 5 μm-thick sections. IHC was conducted following the published protocol.^1^ Briefly, antigen retrieval was achieved by incubating the sections in sodium citrate buffer (pH 6) at 100℃ for 30 minutes. Subsequently, sections were incubated at 4°C overnight with primary antibodies, including anti-GXYLT2 (1:200), anti-β-catenin (1:500, 66379-1-Ig; Proteintech), and anti-Ki67 (1:400, Ab15580; Abcam), diluted in PBS with 5% goat serum. Sections were then treated with the corresponding horseradish peroxidase (HRP)-conjugated secondary antibodies. Visualization of the immunoreactivity was achieved using a DAB visualization kit (DAB-0031; MXB Biotechnologies), followed by counterstaining with hematoxylin. IHC scores were evaluated as the staining intensity (0 = undetectable; 1 = weak; 2 = moderate; 3 = strong) multiplied by the percentage of positive-stained area (1 = 0–33%; 2 = 34–66%; 3 = 67–100%). Antibody information was listed in Table S1.

Cell lines and cell culture

Human GC cell line (AGS, HGC-27, MKN1, MKN45, MKN74, SNU-1, and SNU-5) were obtained from the Cell Bank of Type Culture Collection, Chinese Academy of Sciences (Shanghai, China) and maintained in RPMI-1640 medium (10-040-CV; Corning), supplemented with 10% fetal bovine serum (FBS, Gibco). 293T cell line was cultured in DMEM medium (10-013-CV; Corning) with 10% FBS. The cell lines were incubated at 37 °C with a humidified atmosphere of 5% CO_2_.

Construction of GC cells with stably overexpressed or knockdown GXYLT2

To generate GC cells with stable overexpression or knockdown of GXYLT2, pLVX-GXYLT2-3×FLAG-IRES-puromycin or pLKO.1-shGXYLT2-puromycin was co-transfected with pSPAX2 and pMD2G plasmids into 293T cells using Polyethylenimine (PEI, #408727; Sigma) to generate lentiviral particles, for the following transduction of corresponding GC cells in the presence of 2 μg/ml polybrene (TR-1003; Sigma). Stable cell lines were selected using puromycin at a concentration of 1-2 μg/ml (#58-58-2; Selleck). The shRNA sequences were listed in Table S2.

Sulforhodamine B (SRB) assay

Cell proliferation was assessed by SRB assay. Briefly, 1-4×10^3^ cells/well were plated in 96-well plates and the starting point was set when cells attached to the bottom. As the indicated time points, cells were fixed with 80 μl 10% trichloroacetic acid (TCA) per well and were stained with 5 mg/ml SRB (S1402; Sigma) in 1% acetic acid, followed by the addition of 100-200 μl 10 mM Tris–HCl to dissolve the SRB. The absorbance was measured at 560 nm by a spectrometer (SpectraMax M5e; Molecular Devices).

Transwell assay

Transwell assay was performed using growth factor-reduced matrigel (#354234; Corning) and transwell insert with 8.0 μm pore size (TCS-003-024; BIOFIL). The transwell inserts were pre-coated with 100 μl matrigel (200-300 μg/ml). Three hours later, 1×10^5^ cells (AGS, HGC-27, MKN1, MKN45, or MKN74) were seeded in the upper chamber with 100 μl serum-free medium, while 700 μl medium with 10% FBS was added below the chamber. After 12 hours for AGS cells, 20 hours for HGC-27 cells, 24 hours for MKN1 cells, 72 hours for MKN45 cells, or 72 hours for MKN74 cells, the invaded cells were fixed with 4% formaldehyde and stained with crystal violet staining solution. Images of stained cells underneath the chamber were acquired with a microscope (DM6 B; Leica) and analyzed by ImageJ Software 8.

Sphere formation assay

1-2×10^4^ AGS, HGC-27, MKN1, MKN45, and MKN74 cells were seeded in 6-well ultra-low attachment plates (#3471; Corning) with DMEM/F12 medium containing human recombinant bFGF (10 ng/mL, AF-100-18B; PeproTech) and EGF (10 ng/mL, GMP100-15; PeproTech). After 10-14 days, spheres with the diameter greater than 50 μm were counted.

RNA-seq analysis

Total RNA was isolated following the TRIzol-based RNA extraction method. The gene expression profiling was based on mRNA sequencing using the Illumina NovaSeq Xplus sequencing platform (Majorbio Bio-pharm Technology). The RNA-Seq data has been deposited to Gene Expression Omnibus (GEO) with the accession number GSE267825. The differentially expressed genes (DEGs) between shControl and shGXYLT2 (sh2) of HGC-27 cells were calculated using the DESeq2 method^2^ in TPM (transcripts per million) data format. DEGSeq2 with |Fold change| ≥1.25 and *p* < 0.05 was considered as DEGs. DEGs were submitted for GO enrichment analyses, using the OmicStudiotools (<https://www.omicstudio.cn/tool>).

GSEA^3^ (Gene set enrichment analysis, version 4.1.0) from the Broad Institute was performed on the HALLMARK, C2 PID, C2 REACTOME, C2 KEGG and C5 GOBP genesets (v7.2). The absolute value of normalized enrichment score (NES) > 1, *p* value < 0.05 and FDR < 0.25 were used as the cutoff values.

Total RNA extraction, reverse transcription and quantitative RT-PCR (qRT-PCR)

Total RNA of cultured cells was extracted by TRIzol reagent (#15596018; Thermo Scientific), and reverse transcribed to cDNA with Hifair II 1^st^ Strand cDNA Synthesis SuperMix (#11123ES60; YEASEN). qRT-PCR was performed using ChamQ Universal SYBR qRT-PCR Master Mix (Q711; Vazyme) and detected by LightCycler 96 detection system (BioRad). Each experiment was performed in triplicate. Data were analyzed by 2^-ΔΔCt^ method and fold change was determined using *ACTB* for normalization. Primers for qRT-PCR were listed in Table S2.

Immunofluorescence (IF) staining

1-3×10^5^ cells/well were plated in 6-well plates fitted with special slides for IF, and after the cells were attached to the wall, they were incubated in serum-free medium for 48 hours. Then, cells with the indicated treatments were fixed by 4% paraformaldehyde at room temperature for 15 minutes. The cells were incubated with 0.1% Triton-X100 in PBS for 10 minutes at room temperature, followed by blocking with 5% BSA in TBST for 60 minutes, and then incubated with a mouse anti-β-catenin antibody (1:100, 66379-1-Ig; Proteintech) at 4℃ overnight. Then, goat anti-mouse IgG (H+L) highly cross-adsorbed secondary antibody, Alexa Fluor Plus 488 (1:500, A32723; Invitrogen) was applied at 37 ℃ for one hour. After DAPI (4’,6-diamidino-2-phenylindole) staining, the cells were visualized by a fluorescence microscope. Antibody information was listed in Table S1.

**Co-immunoprecipitation (Co-IP) assay**

8×10^5^ 293T cells were seeded into a 6-cm dish. Twelve hours later, cells were transfected with an empty control, GXYLT2-FLAG, PP2A Aα-HA (8-589)^4^, or GXYLT2-FLAG + PP2A Aα-HA (8-589), respectively. After 72 hours, cells were lysed with NP40 buffer (P0013F; Beyotime) containing complete Protease Inhibitor Cocktail (#4693116001; Roche) and PhosSTOP (#4906845001; Roche). Immunoprecipitation was performed using FLAG- or HA-tag antibodies; 2 µL of antibody added to 1 mg of protein lysates and incubated at 4°C overnight. Samples were then incubated with Protein A/G PLUS-agarose (sc-2003; Santa Cruz) at 4°C for 6 hours. After washing eight times with NP40 buffer containing 1 mM phenylmethanesulfonyl fluoride (PMSF, ST506; Beyotime), samples were used for further Western blotting analyses.

**Phosphatase activity assay**

4.5×10^5^ HGC-27 cells were seeded into a 6-cm dish. After serum starvation for 48 hours, cells were treated with okadaic acid (OA, 30 nM) (S30686; Yuanye Biotechnology) for 8 hours prior to sample collection using NP40 buffer. PP2A activity was measured using malachite green phosphate assay kit (MAK307; Sigma), according to the manufacturer's instructions. The results were normalized to total protein.

Tumorigenicity study

4×10^6^ MKN45 shGXYLT2 cells or mock cells (shC) were subcutaneously injected into the flanks of sixteen-week-old female immunodeficient mice (GemPharmatech). One week after cell inoculation, tumor size and body weight were monitored every 4 days. Tumor volume was calculated using the formula: Volume = Length × Width^2^ × 0.5. All experimental procedures were approved by the Institutional Animal Care and Use Committee of Shanghai Institute of Materia Medica, Chinese Academy of Sciences.

References

1. Chang C, Liu J, He W, et al. A regulatory circuit HP1γ/miR-451a/c-Myc promotes prostate cancer progression. Oncogene. 2018;37:415-426.
2. Love MI, Huber W, Anders S. Moderated estimation of fold change and dispersion for RNA-seq data with DESeq2. Genome Biol. 2014;15:550.
3. Subramanian A, Tamayo P, Mootha VK, et al. Gene set enrichment analysis: a knowledge-based approach for interpreting genome-wide expression profiles. Proc Natl Acad Sci USA. 2005;102(43):15545-15550.
4. Tang Y, Fang G, Guo F, et al. Selective inhibition of STRN3-containing PP2A phosphatase restores hippo tumor-suppressor activity in gastric cancer. Cancer Cell. 2020;38:115-128.
